# EdgeVolution: democratizing multi-objective neural architecture search and end-to-end deployment on microcontrollers

**DOI:** 10.1038/s44172-026-00708-2

**Published:** 2026-06-23

**Authors:** René Groh, Stefan Dendorfer, Mateo Ávila Pava, Fabio Egle, Sebastian Zimmermann, Andreas M. Kist

**Affiliations:** Department Artificial Intelligence in Biomedical Engineering, Friedrich-Alexander-Universität Erlangen-Nürnberg, Erlangen, Bavaria Germany

**Keywords:** Biomedical engineering, Engineering

## Abstract

Edge AI holds great potential for extending the use of artificial neural networks to resource-constrained edge devices, such as microcontrollers. Despite this potential, optimizing and deploying neural networks on these platforms remains challenging due to a lack of tools for hardware-specific adaptation, leading to reproducibility issues and suboptimal performance. To address these challenges, we present *EdgeVolution*, an end-to-end hardware-in-the-loop platform that facilitates multi-objective optimization, neural architecture selection, and direct deployment onto target hardware. We demonstrate the versatility of *EdgeVolution* through four application use cases, showcasing its wide-ranging applicability. By offering a generic and adaptable pipeline, *EdgeVolution* enables the creation and deployment of neural network models tailored to specific datasets, classification tasks, and hardware constraints, thereby improving accessibility, performance, and reproducibility for AI applications on edge devices.

## Abstract (web summary)

René Groh and colleagues report on EdgeVolution, an end-to-end framework for discovering and deploying optimized neural network architectures on microcontrollers. This solution bridges the gap between AI innovation and practical edge computing.

## Introduction

Over the last decade, the need for artificial intelligence (AI) in highly constrained hardware environments such as wearables, ultra-low-power autonomous systems, and IoT devices has increased exponentially, leading to the need for more specialized solutions that are energy-efficient and fast while still being accurate^1^. This paradigm shift towards Edge AI computing has been necessitated by several critical constraints in real-world deployments: (1) inference time (latency) requirements for time-sensitive applications, (2) privacy concerns when transmitting sensitive data, (3) limited network bandwidth and connectivity at the edge, especially in network-challenged environments, and (4) the need for extended operational lifetimes, in some cases even measured in years^2–4^.

A significant challenge in this domain is the widening disparity between the rapid advancement of AI innovations and their practical implementation on resource-constrained edge devices. While breakthrough AI models, applications, and specialized hardware architectures emerge at an unprecedented pace^5–8^, their adoption in edge computing scenarios (Edge AI) remains limited^9^. This implementation gap stems from various technical complexities, including model optimization requirements, hardware compatibility issues, and the expertise needed for efficient deployment. Stakeholders across numerous sectors who could benefit substantially from these advances often find themselves unable to leverage them due to these technical barriers. Consequently, there exists a pressing need for a unifying framework that can seamlessly adapt to emerging neural network architectures and novel hardware platforms, enabling broader accessibility and utilization of cutting-edge AI capabilities, specifically on microcontrollers as a key component in low-power environments.

To address this challenge, there is an increasing trend to combine neural architecture search (NAS) with hardware-aware optimization strategies, formulating it as a multi-objective optimization problem that balances model performance against hardware constraints^10,11^. This approach aims to fully automatically discover neural network architectures that are both accurate and efficiently deployable (inference speed/latency and power consumption) on target edge devices.

Several methods have emerged that can be categorized based on how they incorporate inference speed/latency information in their optimization process. The first category relies on pre-measured lookup tables. FBNet^12^ measures the latency of each operation in the search space and stores these values in a lookup table, estimating total latency by summing operation latencies. Similarly, NetAdapt^13^ creates layer-wise lookup tables with pre-measured resource consumption, including latency, on target platforms. King et al.^14^ and Lyu et al.^15^ follow this approach as well, using pre-investigated lookup tables for operation latency prediction. The second category uses predictive models to estimate latency. ProxylessNAS^16^ employs latency predictors trained on a subset of architectures. OnceNAS^17^ introduces DARTS-Bench (based on DARTS^18^) with hardware-related information and uses a performance predictor trained on this data. The third category relies on proxies instead of actual latency. MicroNets^19^ uses the number of operations as a proxy for latency, guiding the search with constraints on SRAM, eFlash, and, indirectly, latency. MCUNet^20^ follows a similar approach, utilizing a proxy metric (FLOPs distribution) rather than direct latency measurements during NAS optimization.

A few methods incorporate some degree of real hardware feedback throughout the search process. MnasNet^21^ directly measures inference latency by running models on mobile devices, incorporating these measurements into its objective function. Once-for-All Networks^22^ perform quantitative latency analysis on various hardware platforms, including FPGAs, GPUs, and CPUs. EdgeImpulse^23^ measures inference time using hardware timers but does not present a specific NAS algorithm with latency integration.

Critically, none of these frameworks performs continuous on-device deployment during the optimization process to measure both real inference time and power consumption, with hardware-in-the-loop. Even approaches that incorporate some direct measurements do so either before optimization (to build, e.g., lookup tables) or only during final evaluation, rather than throughout the iterative search process. These approximation methods can introduce potentially significant discrepancies between predicted and actual deployment outcomes, particularly when applied to novel neural architectures or emerging hardware platforms (which are also unsupported in most methods when not having a prior, separate latency evaluation). Furthermore, the few frameworks that do include hardware measurements throughout their search process focus primarily on larger platforms like mobile devices, neglecting microcontroller applications. Consequently, while prior works often prioritize low search cost via approximations, our approach deliberately accepts the slightly higher computational overhead of hardware-in-the-loop as a necessary trade-off to guarantee physical deployability and adaptability to novel hardware targets.

Here, we developed *EdgeVolution*, a comprehensive, open-source framework for microcontrollers that bridges the gap between theoretical model optimization and practical edge deployment through direct hardware-in-the-loop evaluation throughout the entire optimization process. *EdgeVolution*, an enhanced framework building upon our prior work “EvoNAS"^24^, addresses the limitations of existing approaches through two key system contributions: (1) the user-friendly implementation of a data-agnostic, multi-objective NAS pipeline that adapts to diverse tasks and modalities, and (2) the integration of real-time, on-device performance measurements—specifically inference speed/latency and power consumption—directly on featured low-power edge devices during the optimization process. By offering a generic and adaptable pipeline, *EdgeVolution* enables the creation and deployment of neural network models tailored to specific datasets, classification tasks, and hardware constraints, improving accessibility, performance, and reproducibility for AI applications on edge devices.

## Results

### A unified framework to enhance edge AI applications

**Fig. 1:**
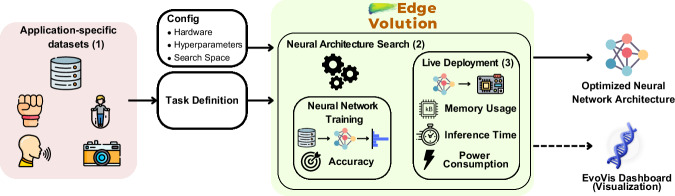
Overview of this study. The generic nature of *EdgeVolution* enables the discovery of highly optimized neural network architectures for different classification tasks and applications. The individual building blocks ((1), (2), and (3)) can be adapted and customized to the user’s needs. Note: The icons in this figure were sourced from Flaticon (https://www.flaticon.com) under a Premium license. These icons are explicitly excluded from the Creative Commons license applied to this article.

*EdgeVolution* is an open-source, end-to-end pipeline—encompassing the entire workflow from initial dataset and hardware definition, through multi-objective NAS incorporating real-time hardware metrics, to the final deployment of an optimized model within a functional binary on the target microcontroller—designed to optimize neural network architectures for low-power microcontroller applications. It is implemented in Python and leverages the Zephyr real-time operating system (RTOS) for direct on-device deployment. The pipeline is divided into three main components, each of which can be independently adapted to meet user requirements: (1) Dataset, (2) NAS, and (3) Zephyr-based Live Deployment (see Fig. 1).

#### Dataset

*EdgeVolution* supports a wide variety of machine learning problems (e.g., classification, regression) by allowing users to create or modify data loaders that inherit from a base class within the framework. This design ensures minimal coding effort while maintaining flexibility in how data is processed for user-specific applications.

#### Neural architecture search

The core objective of *EdgeVolution* is to discover novel neural network architectures that are tailored for a given hardware target. In this study, *EdgeVolution* was executed using our own implementation of an evolutionary search strategy (genetic algorithm) for NAS. However, the framework’s modular code architecture allows other search methods to be integrated seamlessly, such as NSGA-II^25^, LEAP^26^, MENNDL^27^ or the other approaches described in Section 1. The current implementation runs in a ’bare metal’ configuration, but can be extended to interface with additional libraries if desired. The EvoVis dashboard (see Supplementary Fig. S7) can be used to visualize the NAS results during and after optimization^28^.

#### Zephyr-based live deployment

To evaluate candidate architectures, *EdgeVolution* employs a multi-objective fitness function (Equation (1)). This function measures not only the model’s accuracy (obtained from training) but also its performance under real deployment conditions on the target hardware. Each model is automatically integrated into a Zephyr-based C++ project, which may already include other crucial application elements such as sensor data acquisition, multithreading, or Bluetooth communication. By incorporating these real-world constraints, the pipeline ensures that the final neural network architecture is truly optimized for practical use and deploys “out of the box." This integration guarantees that the resulting model meets all hardware limitations and application-specific requirements without further manual intervention.

### NAS for optimizing neural network performance on microcontrollers

**Fig. 2:**
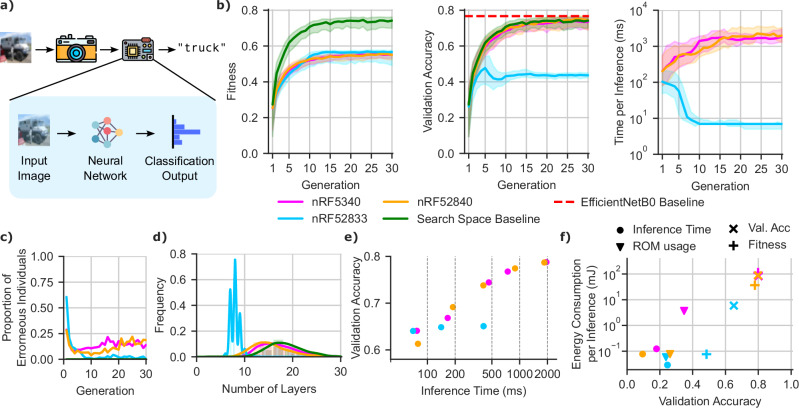
*EdgeVolution* results on the Cifar-10 dataset. **a** Overview of the image classification task. **b** Median fitness, validation accuracy and measured time per inference across evolutionary generations, with the shaded area representing the 25th to 75th percentiles. The search space baseline follows the defined search space but excludes microcontroller constraints. **c** Proportion of erroneous individuals in each generation for each microcontroller target. Neural networks that violate the implicit constraints of a microcontroller target—such as exceeding RAM limits (see Section 1)—are classified as erroneous, as they are not deployable. Consequently, objective measures like inference time and energy consumption cannot be evaluated for these individuals. **d** Distribution of the number of layers in neural networks sampled from the search space, considering only individuals within the top 5% fitness across all generations. **e** Best achieved validation accuracies of individual neural networks from each optimization target, filtered by different inference time thresholds. For the nRF52833 microcontroller, no data points exist at higher inference time thresholds due to deployment constraints—neural networks exceeding these limits are too large to be deployed. **f** Validation accuracy versus energy consumption per inference for the best-performing individuals in each objective for all three microcontroller optimization targets. Note: the icons in this figure were sourced from Flaticon (https://www.flaticon.com) under a Premium license. These icons are explicitly excluded from the Creative Commons license applied to this article.

To validate the effectiveness of *EdgeVolution*, we applied our evolutionary NAS to the CIFAR-10 dataset. This initial experiment demonstrates our approach’s capability to adapt neural network architectures for microcontroller deployment while optimizing multiple objectives. The classification task is illustrated in Fig. 2a, and further methodological details are provided in Section 1.

The optimization process was executed using the hyperparameters described in Section 1, including a total of 30 generations, a number empirically determined as a balanced trade-off between convergence and computational cost. Due to the population decay strategy (see Supplementary Fig. S9), a total of 4600 architectures were sampled and evaluated per optimization run, each run being repeated three times for statistical robustness. A detailed breakdown of the search time and computational cost is provided in Supplementary Fig. S4, demonstrating that the hardware-in-the-loop evaluation accounts for less than 1% of the total runtime.

The evolutionary process demonstrates a notable fitness convergence across different microcontroller targets. As shown in Fig. 2b, the fitness function effectively guides optimization, leading to stable fitness values ~0.5 for all microcontroller targets. This represents a midpoint in our 0-to-1 fitness scale, where 1 would indicate a perfectly optimized neural network architecture with optimal values for all objectives. It is important to note that achieving a fitness of 1 is deliberately challenging, as we selected optimal objective values that are unlikely, if not impossible, to be simultaneously achieved by a single solution. The optimization follows the Pareto-front principle, i.e., a neural network architecture is considered optimal if no objective can be improved without degrading at least one other objective. The Pareto-front is further explained in Section 1. Additionally, we trained an EfficientNetB0^29^ to determine the baseline accuracy that can be reached by a neural network architecture with a large parameter space that is thus also not deployable to hardware-constrained environments.

The search space baseline, which excludes hardware deployment constraints, achieves slightly higher fitness values because it only considers validation accuracy rather than the full set of deployment constraints. Despite incorporating microcontroller constraints for all optimization targets, validation accuracy remains comparable between microcontroller-specific models and the search space baseline, as illustrated in Fig. 2e. Notably, models optimized for nRF52840 and nRF5340 achieve accuracy levels approaching the EfficientNetB0 baseline, while the more resource-constrained nRF52833 shows lower performance. These results demonstrate that our models achieve strong accuracy relative to established benchmarks, even though they do not approach the theoretical maximum fitness of 1.0. The fitness is necessarily limited by two factors: first, the inherent difficulty of achieving perfect accuracy on any dataset itself, and second, the unavoidable tradeoffs between optimizing resource usage (ROM and energy consumption) and maintaining high accuracy when targeting resource-constrained microcontrollers.

Inference time is a crucial aspect of real-world deployment on microcontrollers, and Fig. 2b highlights how *EdgeVolution* optimizes models differently depending on the hardware constraints. The nRF52840 and nRF5340 converge towards an inference time of approximately 1000 ms, while the nRF52833 reaches a lower inference time of around 10 ms. This demonstrates the effectiveness of the fitness function in adapting network size and complexity to ensure real-time deployability. Moreover, Fig. 2e shows that increasing the inference time budget allows larger microcontrollers to achieve an improved validation accuracy, whereas for nRF52833, no such improvement is observed. This is due to deployment constraints hindering larger models from running on the hardware, emphasizing the implicit limitations imposed by microcontroller-specific constraints.

Some architectures become undeployable due to exceeding hardware limitations, such as RAM or ROM constraints. As demonstrated in our analysis (Supplementary Fig. S1), proxy metrics such as operation count exhibit a weak correlation with actual inference time on microcontrollers and fail to distinguish between these deployable and non-deployable architectures (e.g., those causing RAM overflows). To quantify the necessity of hardware-in-the-loop evaluation, we performed an ablation study comparing our approach against a proxy-based optimization using operation counts (see Supplementary Fig. S2). The results demonstrate that proxy-based search tends to collapse towards minimal model sizes, failing to exploit the available hardware resources effectively. Only our strategy successfully explored the upper Pareto-front (high accuracy, acceptable energy), whereas the proxy approach blindly optimized for low complexity, missing the optimal trade-off between performance and resource usage. By relying on hardware-in-the-loop, *EdgeVolution* incorporates these limitations as an implicit constraint within the optimization framework. This implicit filtering mechanism ensures that our optimization not only addresses theoretical performance metrics but also guarantees practical deployability in resource-constrained environments. Figure 2c illustrates the proportion of erroneous individuals during the optimization process. At the beginning of the runs, 50% of the individuals in nRF52833 and 25% in nRF52840 and nRF5340 are considered erroneous, as they fail deployment due to resource constraints. Over successive generations, these numbers decrease, indicating that the search process successfully reduces non-deployable architectures. This reduction occurs naturally through the evolutionary algorithm’s selection mechanism: only networks that are deployable serve as ancestors for the next generation. As a result, the algorithm progressively narrows its focus to the viable solution space, efficiently eliminating architectures that exceed hardware constraints while preserving and refining those that balance performance with deployability requirements. However, for nRF52840 and nRF5340, the proportion of erroneous individuals rises again in later generations. This suggests that *EdgeVolution* explores the search space to the point where small mutations push previously deployable architectures beyond hardware limits.

A deeper look into the architectural properties of the best-performing models reveals clear hardware-specific trends. Figure 2d illustrates that models optimized for nRF52840 and nRF5340 tend to have deeper architectures with more layers, balancing accuracy and compactness. In contrast, the nRF52833 predominantly samples networks with a maximum depth of around ten layers, emphasizing efficiency and speed over complexity. This architectural trend aligns with the hardware constraints of the respective microcontrollers, highlighting the adaptability of *EdgeVolution’s* search strategy. Figure 2f illustrates the trade-offs between accuracy, energy consumption, and ROM usage. The most energy-efficient models (dots) and those with the lowest ROM usage (triangles) consistently achieve lower accuracy, while the most accurate architectures (top-right corner) demand higher energy per inference. Among the optimized models, the nRF52833 achieves a well-balanced compromise between accuracy and energy efficiency, whereas the nRF52840 and nRF5340 prioritize accuracy at the cost of increased energy consumption. This result highlights the strong influence of fitness function weighting, which in this case heavily favors accuracy maximization, as reflected by the weighting factor of 0.7 (see Section 1). Adjusting these weights would allow the optimization to shift towards lower-power solutions, but this would inevitably come at the expense of accuracy, as trade-offs along the Pareto-front must be considered.

Our results clearly indicate that *EdgeVolution* effectively optimizes neural network architectures for use on resource-constrained microcontrollers by utilizing on-device deployment. We found that the evolutionary NAS successfully identifies architectures that balance accuracy, inference time, and energy efficiency while meeting implicit deployment constraints.

### Achieving high-accuracy keyword spotting on resource-constrained hardware

**Fig. 3:**
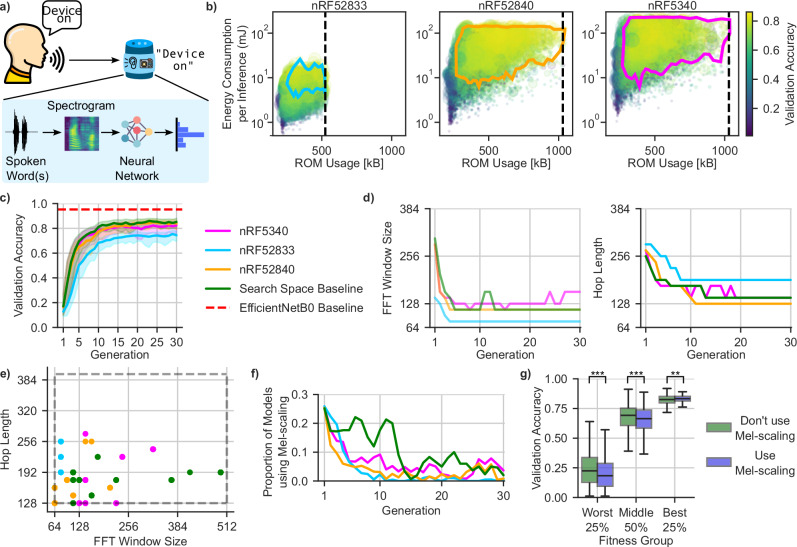
*EdgeVolution* results on the Speech Commands dataset (same color-code as in Fig. 2). **a** Overview of the keyword spotting task. **b** Pareto fronts for each microcontroller optimization target, where each point represents a neural network architecture. Point sizes indicate generation, with smaller points corresponding to earlier generations. The regions for each microcontroller represent the alpha shape encompassing all architectures with a validation accuracy above 0.8. **c** Median validation accuracy across evolutionary generations, with the shaded area representing the 25th to 75th percentiles. The search space baseline follows the defined search space but excludes microcontroller constraints. **d** Dominant FFT window size and hop length selection for STFT calculation during evolutionary optimization across generations. **e** Dominant combinations of FFT window sizes and hop lengths for STFT calculation, considering only the top 10% of performers based on fitness values. Dashed lines indicate the search space boundaries. **f** Usage of Mel-scaling across generations. **g** Impact of Mel-scaling on neural network performance. Neural architectures from all optimization targets were categorized into three fitness groups (Worst 25%: *n* = 8094, with 6686 without Mel-scaling and 1408 with Mel-scaling; Middle 50%: *n* = 16,186, with 15,420 without Mel-scaling and 766 with Mel-scaling; Best 25%: *n* = 8094, with 7893 without Mel-scaling and 201 with Mel-scaling). Performance differences between architectures with and without Mel-scaling were assessed using a two-tailed Mann-Whitney U-test due to non-normally distributed data (see Supplementary Table S2). Asterisks indicate statistical significance following Bonferroni correction for three comparisons (*p* < 0.017: *, *p* < 0.003: **, *p* < 0.0003: ***). Note: the icons in this figure were sourced from Flaticon (http://www.flaticon.com) under a Premium license. These icons are explicitly excluded from the Creative Commons license applied to this article.

A common task for edge devices is the processing and machine learning-based analysis of one-dimensional, longitudinal data, such as audio. To assess the performance of *EdgeVolution* on audio-based tasks, we applied our evolutionary NAS approach to the Speech Commands dataset, a widely used benchmark for keyword spotting. This experiment evaluates the capability of our approach to optimize both neural network architectures and audio preprocessing parameters while considering microcontroller constraints. The classification task setup is depicted in Fig. 3a.

The available ROM strongly influences the search space and feasible architecture configurations. The nRF52833, with only 512 kB of ROM, is constrained to a much smaller set of architectures compared to the nRF52840 and nRF5340, which each have 1024 kB of ROM. This difference is reflected in the distribution of sampled neural network architectures, where energy-efficient models appear in the lower region of the Pareto plots, but at the cost of validation accuracy (Fig. 3b). This highlights the inherent trade-off between resource-efficiency and accuracy, emphasizing that the final model selection depends on application-specific requirements and can be fine-tuned in post-processing.

The accuracy achieved by the microcontroller-optimized models is lower than the EfficientNetB0 baseline, which was also trained for the keyword spotting task and serves as an upper bound for performance (Fig. 3c). The search space baseline, which does not enforce hardware constraints, achieves slightly lower accuracy than EfficientNetB0 but remains undeployable on microcontrollers. Among deployable models, nRF52840 and nRF5340 achieve the highest accuracy, whereas nRF52833 lags behind, with a median validation accuracy between 0.75 and 0.8. This discrepancy results from the more restrictive memory and computational resources available on the nRF52833.

Audio preprocessing choices have an impact on performance. The short-time Fourier transform (STFT) converts the raw audio waveform into a two-dimensional time-frequency spectrogram by computing the discrete Fourier transform over overlapping time windows. Two key parameters govern this transform: the FFT window size, which determines the frequency resolution, and the hop length, which controls the temporal stride between consecutive windows. Optionally, the resulting linear-frequency spectrogram can be mapped onto the Mel scale, a perceptually motivated frequency axis that compresses higher frequencies where human auditory sensitivity is lower. While STFT parameters and Mel scaling are commonly treated as fixed design choices in speech processing pipelines, we included them in our search space to evaluate their interaction with neural architecture design under microcontroller constraints. Selecting optimal preprocessing parameters is critical: overly high resolution increases RAM usage beyond available limits, whereas overly low resolution degrades classification accuracy.

The optimization consistently favors smaller FFT window sizes, with a median around 128, despite the common expectation of larger values around 1024 in the literature (Fig. 3d). Hop length values predominantly fall between 128 and 256, with the nRF52833 favoring slightly higher values. This is likely due to its lower RAM capacity (128 kB compared to 256 kB and 512 kB for the other microcontrollers), which constrains feasible preprocessing parameters.

The selection of top-performing models further reinforces these trends. Most optimized models, regardless of hardware target, prefer smaller FFT window sizes and hop lengths between 128 and 256 (Fig. 3e). Only the largest microcontroller, nRF5340, and the search space baseline occasionally favor larger FFT window sizes up to 512. This suggests that even when larger FFT windows are possible, they are rarely selected during optimization, indicating that they are suboptimal w.r.t. the fitness function.

Next, we analyzed the use of Mel-scaling, a commonly used preprocessing step in speech recognition. Its usage declines steadily over generations, dropping below 10% (Fig. 3f). Moreover, architectures that incorporate Mel-scaling consistently exhibit lower validation accuracy across different fitness groups (Fig. 3g). This contradicts conventional expectations, as Mel-spectrograms are widely used in speech processing pipelines. However, these results indicate that, within the specific search space and optimization framework of *EdgeVolution*, Mel-scaling does not always seem to enhance performance. This finding underscores the ability of automated architecture search to uncover design choices that may be overlooked in traditional manually tuned approaches.

Using the Speech Commands task, we also evaluated the extensibility and computational demands of our framework. First, to highlight the framework’s modularity, we integrated four alternative search methods (Regularized Evolution^30^, NSGA-II^25^, Bayesian Optimization^31^, and Random Search)). A brief evaluation on the Speech Commands dataset (Supplementary Fig. S3) indicates that our custom genetic algorithm provides a strong baseline, achieving competitive validation accuracy and effectively exploring the architectural search space alongside these established methods. Furthermore, we analyzed the computational cost of the search process. In this study, *EdgeVolution* trained each candidate model to ensure reliable performance assessment. As shown in our search time analysis (Supplementary Fig. S4), training accounts for 99% of the total optimization time, whereas the actual on-device measurement is comparatively negligible. While this approach guarantees evaluation accuracy, it also constrains the framework’s applicability to small and medium-sized datasets. As an alternative to reduce search cost, we implemented two complementary approaches: (1) surrogate models that predict validation accuracy or hardware energy consumption from architecture encodings, allowing the search to skip training or hardware evaluation for low-confidence candidates (Supplementary Fig. S5), and (2) hardware lookup tables trained from an initial profiling run, which approximate energy and inference time without requiring a physical hardware connection during the search (Supplementary Fig. S6). Finally, we successfully demonstrated the easy expansion to diverse hardware targets via Zephyr RTOS integration by performing an optimization for the STM32H753ZI microcontroller on this dataset (see Supplementary Fig. S12).

### Real-time and accurate hand posture classification on low-power microcontrollers

**Fig. 4:**
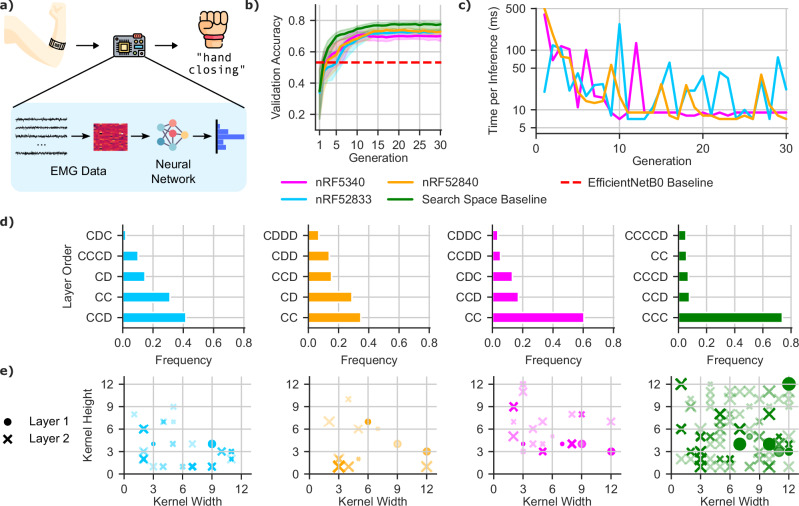
*EdgeVolution* results on the sEMG dataset (same color-code as in Fig. 2). **a** Overview of the hand posture classification task. **b** Median validation accuracy across evolutionary generations, with the shaded area representing the 25th to 75th percentiles. The search space baseline follows the defined search space but excludes microcontroller constraints. **c** Inference time per generation for the best-performing neural network architecture based on validation accuracy. **d** Relative frequency of convolutional (C) and depthwise convolutional (D) layer orderings at generation 30, showing only the top five most common sequences. **e** Kernel width and length for the first (dots) and second (crosses) layers, filtered by the most preferred convolutional and depthwise layer order and the top 10% of architectures based on fitness. Marker size reflects the number of filters used in each layer, with larger markers indicating more filters. Note: the icons in this figure were sourced from Flaticon (https://www.flaticon.com) under a Premium license. These icons are explicitly excluded from the Creative Commons license applied to this article.

Human-in-the-loop applications often require machine learning solutions to provide real-time control and feedback. One example in this context is the usage of surface electromyography (sEMG) signals to control prostheses with multiple degrees of freedom (DoFs). To highlight the capabilities of *EdgeVolution* to cope with sEMG data streams and achieve real-time classification results, we ran experiments to classify different hand postures (see Fig. 4a).

The neural architectures optimized by *EdgeVolution* achieve competitive performance compared to the EfficientNetB0 baseline after several generations of evolutionary optimization (Fig. 4b). This could potentially be attributed to the relatively small dataset, which may favor our compact architectures. While this limits absolute accuracy, the task itself sufficiently demonstrates real-time processing capabilities on resource-constrained devices—our primary objective. Future work can improve accuracy by expanding the training data. Additionally, our search space encourages architectures better suited to sEMG signal characteristics, enabling more efficient feature extraction.

Inference time, particularly for the best-performing models in each generation, demonstrates a decreasing trend throughout the optimization process (Fig. 4c). However, the extent of stabilization varies across microcontroller targets. For the nRF52833, inference time fluctuates between 10 ms and 100 ms due to mutations introducing architectural variations. The nRF52840 exhibits a similar oscillatory behavior but within a narrower range of 10–50 ms. In contrast, the nRF5340, being the most computationally powerful microcontroller, consistently maintains inference times below 10 ms, leading to more stable results. These differences highlight the difficulty of balancing the trade-off between accuracy and inference time for less powerful microcontrollers, where optimizations must account for stricter resource constraints. In the context of prosthesis control, an overall delay of 100–300 ms is regarded as an acceptable real-time control for the user^32,33^. Taking into account the window size of 100 ms, the proposed system could therefore offer the prosthesis user a smooth control without a noticeable delay^34^.

To further analyze the optimized architectures, we examined the top-performing models at the final generation for each optimization target. Across all microcontrollers, architectures with two or three convolutional layers for feature extraction were consistently favored, suggesting that this depth is well-behaving for extracting relevant information from the sEMG signals (Fig. 4d).

A deeper analysis of kernel dimensions reveals a distinction between kernel height and width across microcontroller targets (Fig. 4e). The results indicate that all microcontroller-optimized models favor kernel heights of 9 or lower, suggesting that compact filter sizes are sufficient for extracting spatial patterns in the input data. However, kernel widths exhibit a much wider distribution across the entire search space, with the most optimized architectures favoring higher values. This trend suggests that capturing temporal dependencies in sEMG signals benefits from increased kernel width, which extends the receptive field across the time axis. As the available computational resources increase, such as in the search space baseline, kernel sizes shift toward larger values, as seen in the top-right region of the plots.

For the search space baseline, no clear trend in kernel sizes emerges, suggesting that the evolutionary process does not exhibit a strong preference when constraints are removed. This lack of convergence may indicate that the fitness function alone does not inherently favor larger receptive fields unless constrained by hardware-specific trade-offs.

### Low-power optimization for on-device IMU-based real-time activity detection

**Fig. 5:**
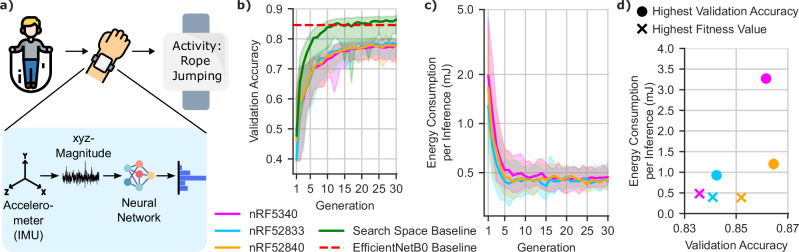
*EdgeVolution* results on the DaLiAc dataset (same color-code as in Fig. 2). **a** Overview of the activity recognition task. **b** Median validation accuracy across evolutionary generations, with the shaded area representing the 25th to 75th percentiles. The search space baseline follows the defined search space but excludes microcontroller constraints. **c** Median energy consumption per inference across evolutionary generations, with the shaded area representing the 25th to 75th percentiles. **d** Validation accuracy versus energy consumption per inference for the best-performing neural networks, selected from all evaluated architectures based on validation accuracy (dots) and achieved fitness value (crosses). Results are presented for three different microcontrollers. Note: The icons in this figure were sourced from Flaticon (https://www.flaticon.com) under a Premium license. These icons are explicitly excluded from the Creative Commons license applied to this article.

Next, we investigated the use of *EdgeVolution* for low-power applications. As an example, we used IMU-based activity detection designed to run continuously in the background on wearables while still detecting activities in real-time. This capability is essential for devices like smart watches, where power efficiency is critical. Figure 5a shows the classification pipeline, in which the magnitude of accelerometer data is fed into a neural network to detect activities such as rope jumping.

The validation accuracy on the DaLiAc dataset steadily increases over the course of evolutionary optimization for all microcontroller targets, although none of these targets reaches the accuracy level of the EfficientNetB0 baseline (Fig. 5b). In contrast, the search space baseline, which does not include microcontroller constraints, not only attains but even exceeds the EfficientNetB0 performance. This highlights that the chosen search space is capable of generating neural network architectures that reach a high classification performance on the human activity recognition classification task.

Energy consumption, a crucial metric for wearable and embedded devices, shows a clear downward trend over successive generations (Fig. 5c). By the final generations, the median energy consumption per inference reaches ~0.5 mJ. For context, Lattanzi et al.^35^ reported energy consumption between 1 mJ and 2.4 mJ on a Cortex-M4 microcontroller smartwatch for a similar eight-class human activity recognition task. These results demonstrate that *EdgeVolution*-generated architectures achieve state-of-the-art energy efficiency levels. Even when selecting neural network architectures with higher validation accuracy, which naturally increases energy consumption (Fig. 5d), our models remain comparable to reference results in terms of energy efficiency.

## Discussion

By merging several key components for high-performance Edge AI applications, *EdgeVolution* provides an important step towards democratizing their development beyond the community of computer science experts. The lower cost and better accessibility of smaller hardware, compared to larger inference platforms such as GPUs, enable our framework to accelerate the adoption of AI models in various domains. Our objective is to make optimization for AI tasks on low-power, small hardware available to a broad range of users.

*EdgeVolution* aims to bridge the gap between novel neural network architectural designs and emerging edge-hardware developments. Existing platforms face notable limitations in this regard. Several prominent approaches—ProxylessNAS^16^, MnasNet^21^, FBNet^12^, Once-for-All Networks^22^, and NetAdapt^13^—target mobile devices and smartphones rather than the more resource-constrained class of microcontrollers. Others, such as OnceNAS^17^, MicroNets^19^, and MCUNet^20^, lack integration of real-world measured inference speed and power consumption. Platforms like EdgeImpulse^23^ rely on numerous predefined assumptions that limit adaptability and customization. *EdgeVolution* addresses these limitations by offering a customizable and practical hardware-software solution, distinguished by three characteristics: (1) hardware deployment and evaluation of candidate neural network architectures during NAS optimization, (2) full open-source availability with customization instructions, and (3) easy expansion to diverse hardware targets via Zephyr RTOS integration, as demonstrated by the successful incorporation of the STM32H753ZI microcontroller.

Among existing approaches, MCUNet^20^ is the most closely related to *EdgeVolution* and warrants a more detailed comparison. Although MCUNet’s TinyEngine demonstrates performance advantages over TFLite Micro and CMSIS-NN, it lacks their widespread integration in established development ecosystems. Our deliberate choice to leverage industry-standard frameworks facilitates immediate deployability within existing workflows, particularly through seamless integration of TFLite Micro into Zephyr RTOS. This design philosophy supports a workflow in which embedded application features are developed first, and neural network optimization is performed as a final step, when available resource constraints—particularly RAM and ROM—are precisely known within an embedded project.

The design of *EdgeVolution* involves several deliberate trade-offs whose implications merit discussion. Our deployment strategy performs on-device hardware evaluation for every candidate model to ensure reliable performance assessment. While thorough, this approach increases search cost relative to methods that rely on predictive modeling of inference metrics. We regard this as a principled choice: as demonstrated by our ablation study, relying solely on proxy metrics—such as operation counts—fails to effectively exploit available hardware and misses optimal performance trade-offs. Predictions of hardware behavior, moreover, often lack generalizability across diverse hardware targets. *EdgeVolution* therefore prioritizes direct hardware-in-the-loop optimization with empirical measurements, accepting increased search cost to guarantee immediately usable neural networks paired with accurate performance characterizations.

A related consequence is that the model training phase constitutes the vast majority of the optimization time, acting as a bottleneck for larger datasets. Our initial implementations of surrogate models and hardware lookup tables demonstrate the feasibility of predicting candidate viability and reducing this training burden. However, we have not yet incorporated zero-shot or one-shot evaluation approaches^36–38^, which have demonstrated considerable efficacy in reducing computational cost in NAS contexts. The integration of such methods represents a promising direction for future development. Should these methods resolve the training bottleneck, the deployment phase would become the dominant cost. To address this, we propose a Success-Driven Quota Deployment strategy. Unlike a rigid ‘top-k’ selection based on accuracy—which risks selecting only large architectures that fail deployment due to memory constraints—this approach iterates through the population ranked by validation accuracy and attempts deployment sequentially until a predefined quota (*N*) of successfully deployed models is reached. These hardware-verified individuals then serve as the parent population for the next-generation, ensuring that optimization prioritizes high-performing models while dynamically filtering out non-deployable architectures.

The classification performance of discovered architectures is at times outperformed by state-of-the-art results. This gap arises from the trade-offs inherent in optimizing neural networks for tiny, low-power hardware. The search space of *EdgeVolution* is constrained by the operations supported in TFLite Micro, which, while comprehensive, may limit exploration of certain architectural innovations. Additionally, our current implementation does not integrate dataset-specific augmentation techniques into the standard pipeline, although such methods could substantially enhance model performance. These constraints should be weighed against the practical advantage that all reported architectures are verified to run on the target hardware—a guarantee that purely accuracy-driven approaches do not provide. Incorporating a modular augmentation framework that adapts to diverse data characteristics could narrow this gap in future releases.

The current multi-objective fitness function employs an empirically-derived weighting scheme that prioritizes accuracy (*w*_A_ = 0.7) over memory (*w*_M_ = 0.1) and energy efficiency metrics (*w*_E_ = 0.2). This reflects the observation that, in resource-constrained environments, secondary objectives often have natural upper bounds imposed by hardware limitations: once a model fits within memory and meets energy requirements, further gains along these axes yield diminishing returns compared to improvements in accuracy. Future implementations could incorporate adaptive weighting schemes that automatically balance multiple objectives based on application profiles.

Finally, the framework’s modularity allows for extension beyond standard microcontrollers. Future iterations could adapt the hardware-in-the-loop pipeline to emerging neuromorphic and in-sensor computing platforms, enabling automated co-design of spiking neural networks or specialized hardware accelerators. As these next-generation chips become more accessible, *EdgeVolution* can serve as a unifying platform to benchmark and optimize their efficiency in real-world edge scenarios.

Altogether, *EdgeVolution* represents a framework that makes Edge AI technology accessible and practical for a diverse user base, bridging a critical gap in edge computing by offering an end-to-end solution that adapts to a wide range of applications. The framework’s modular architecture ensures continual alignment with ongoing developments: as the open-source Zephyr real-time operating system evolves through community contributions, *EdgeVolution* directly benefits from these improvements, and advances in NAS can be readily incorporated. Together with the support of the Edge AI community, we aim to further develop and improve *EdgeVolution* as an accessible open-source framework for end-to-end NAS on resource-constrained edge devices.

## Methods

### NAS and multi-objective optimization

A central aspect of this study is the optimization of neural network architectures to meet real-world requirements. To achieve this, a NAS is utilized. NAS can be broadly divided into three components: the search space, the search strategy and the estimation strategy^39^.

#### Search space

The search space defines the set of possible neural network architectures and can be constructed in various ways, such as macro search space, chain-structured search spaces or cell-based search spaces^40^. In this study, the search space is detailed as part of the hyperparameter configuration in Section 1.

#### Search strategy

The search strategy determines how candidate architectures are explored within the search space. Common search strategies include reinforcement learning^21,41^, Bayesian optimization^31,42,43^, gradient-based search^18^, and evolutionary optimization^44,45^. While all of these strategies could potentially be integrated into the *EdgeVolution* pipeline, we employ our own implementation of evolutionary optimization as part of the first release. However, to demonstrate our code-modularity, we implemented four additional strategies (Regularized Evolution^30^, NSGA-II^25^, Bayesian Optimization^31^, and Random Search), the performance of which is evaluated in Section 1.

Specifically, we utilize a genetic algorithm to discover neural architectures tailored to the requirements and constraints of low-power hardware. The process begins with sampling random architectures (n_initial_population_) from the search space to initialize the first generation. Each of these architectures is evaluated using the estimation strategy, resulting in a fitness value between 0 and 1.

**Fig. 6:**
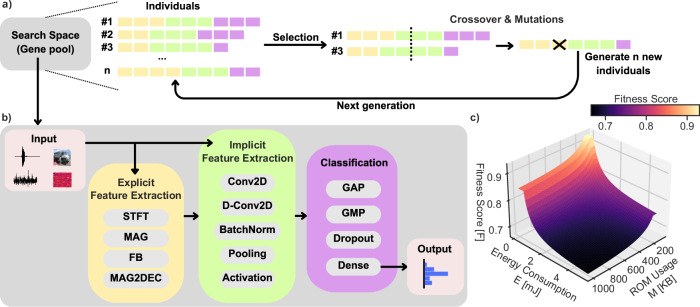
Evolutionary optimization of neural network architectures for microcontrollers. **a** Workflow of the evolutionary algorithm, including selection, crossover, and mutations to generate new architectures for each new generation of individuals. **b** Neural network architecture pipeline from input feature extraction to classification and output, forming the basis of the search space used in this work. **c** Fitness score landscape visualized as a function of energy consumption and ROM usage. Darker regions indicate lower fitness scores, calculated using Equation (1) with accuracy constrained at 0.9. By fixing accuracy at this threshold value, we isolate and examine only solutions that meet or exceed 90% classification accuracy, creating a 2D slice through the full 3D objective space. The landscape illustrates how the fitness function can navigate the objectives to maximize fitness by minimizing energy consumption and ROM usage of the neural network architectures.

Based on the fitness values, individuals are selected to form the next generation. While various selection techniques are available^46^, we employ a combination of truncation selection and roulette wheel selection in this study. Initially, the best architectures (*n*_*selection*_) are chosen based on their fitness values. These selected architectures are then sorted and assigned probabilities based on a uniform distribution. Using this subpopulation and their assigned probabilities, one-point crossover is performed to generate a new population of candidate architectures. Each newly generated architecture is then subjected to mutation with a predefined mutation probability. This entire process is depicted in more detail in Fig. 6.

#### Estimation strategy

The estimation strategy is closely related to the concept of objective functions in optimization, as it evaluates the performance of a given candidate neural network architecture. For embedded devices, this involves addressing a multi-objective optimization problem^47^ where multiple real-world objectives must be considered simultaneously. Each real-world parameter is represented as a complementary objective function *f*_1_, …, *f*_*k*_, forming a space *N* of feasible solutions, where *N* represents the candidate neural network architectures. In most cases, there is no single solution N^*^ ∈ *N* that optimizes all objective functions *f*_1_, …, *f*_*k*_ simultaneously. Instead, there are tradeoffs between objectives, where improving one objective *f*_*i*_ leads to a tradeoff in at least one other objective *f*_*j*_. Solutions that balance these trade-offs are called Pareto-optimal solutions, and the set of all Pareto-optimal solutions forms the Pareto-front.

In our work, we define explicit and implicit objectives. Explicit objectives include classification accuracy (or some other defined performance metric), ROM (flash memory) usage on the microcontroller, and energy consumption, which includes inference time (Section 1) and power consumption (Section 1). Implicitly, the inference strategy is influenced by the amount of RAM available on the microcontroller, since this parameter determines the size of the tensor arena and thus the size of the models. Candidate architectures that exceed the RAM constraints are considered infeasible and are assigned a performance estimation value of zero. In this study, the performance estimate is referred to as the fitness (section 1), in keeping with the terminology commonly used in evolutionary optimization processes. We deliberately avoid the use of proxy metrics (e.g., FLOPs) as objective functions. Our empirical data show that such proxies are insufficient for characterizing the non-linear resource constraints of microcontroller hardware (see Supplementary Fig. S1), often leading to the selection of invalid architectures.

### Hardware

In the following, we introduce the target hardware used in this study. Each hardware component in our framework is designed to be interchangeable, providing users the flexibility to incorporate and utilize hardware other than that presented here, tailored to their specific requirements. Furthermore, we describe the deployment process and the relevant software packages included to enable on-device inference, which is one aspect of our multi-objective optimization workflow.

#### Microcontrollers

For our study, we utilized three microcontrollers with varying hardware configurations to demonstrate the capability of *EdgeVolution* to adapt to different memory, processing, and hardware constraints (Supplementary Fig. S10 shows the hardware setup). The selected microcontrollers were the nRF5340, nRF52840, and nRF52833 from Nordic Semiconductor. The nRF5340 features a 64 MHz Arm Cortex-M33 application processor with 1 MB of Flash memory and 512 KB of RAM. The nRF52840 includes a single-core 64 MHz Arm Cortex-M4 processor, alongside 1 MB of Flash memory and 256 KB of RAM. Similarly, the nRF52833 is powered by a 64 MHz Arm Cortex-M4 processor, with 512 KB of Flash memory and 128 KB of RAM. All used microcontrollers also include a floating-point unit (FPU).

#### Power consumption measurement

We utilized the Power Profiler Kit 2 (PPK2) from Nordic Semiconductor, which is compatible with all the microcontrollers introduced earlier. The device was configured in ampere meter mode with a source voltage of 3.3 V, and the “device under test" (DUT) option was enabled. Measurements were recorded at a frequency of 100,000 samples per second. To process the data and obtain the mean power consumption, a rolling average with 1 ms windows was applied, followed by thresholding to identify the start and end of each inference cycle. The energy consumption *E* for one inference cycle is calculated using the voltage, the mean power consumption during the inference cycle, and the measured inference time. This calculation includes the baseline power consumption of the microcontroller in idle mode. Given a fixed voltage of 3.3 V, the energy consumption in millijoules (mJ) is computed as:

$$E=V\cdot \left({I}_{{{\rm{mean}}}}+{I}_{{{\rm{idle}}}}\right)\cdot {t}_{{{\rm{inference}}}},$$

where *V* = 3.3 *V* is the source voltage, *I*_mean_ is the mean current consumption (in amperes) during the inference cycle, *I*_idle_ is the baseline current consumption of the microcontroller in idle mode (in amperes), and *t*_inference_ is the inference time (in seconds). To compute energy consumption, the mean power consumption during one inference cycle is determined. An example of the measurement of one inference cycle is shown in Supplementary Fig. S11.

### Software

*EdgeVolution* connects several open-source software libraries to offer an end-to-end software chain for optimizing and deploying artificial neural networks.

#### TensorFlow und TensorFlow Lite Micro

We utilize TensorFlow (version 2.9) as part of the *EdgeVolution* framework. TensorFlow provides a robust platform for performing deep learning tasks, including the creation, training, and validation of artificial neural networks. It is optimized for GPU integration, offering an efficient and fast way to train neural networks. Within the *EdgeVolution* framework, all candidate neural networks are trained using TensorFlow.

For edge deployment, however, TensorFlow is unsuitable for resource-constrained devices like microcontrollers. This limitation arises due to TensorFlow’s lack of resource-aware implementations of operations, incompatibility with microcontroller runtime environments, and its high memory footprint. To address these challenges, the TensorFlow Lite Micro (TFLite Micro) environment, also known as LiteRT for microcontrollers, is used to deploy neural networks on edge devices.

To leverage TFLite Micro, TensorFlow models must be converted into a lightweight format suitable for microcontroller execution. Only a subset of TensorFlow operations is supported in TFLite Micro, including basic operations such as addition, multiplication, convolution, pooling, and activation functions (e.g., ReLU, sigmoid). Consequently, the architectures of neural networks must be constrained to use only the operations (op sets) available in TFLite Micro. In our work, we utilize the TFLITE_BUILTINS op set, which includes core operations optimized for TFLite, and the SELECT_TF_OPS op set, which allows the use of certain TensorFlow operations not natively supported in TFLite (overview of available TFLite Micro ops in Supplementary Table S1). Additionally, we leverage the CMSIS-NN package^48^, which provides highly optimized neural network kernels specifically designed for Arm Cortex-M processors. By integrating CMSIS-NN, we further enhance the inference efficiency of our deployed models.

In the case of edge devices that require further optimization, such as a reduction in memory usage and an increase in inference speed, the TFLite converter offers the option of quantization. In our study, we applied integer-only (INT8) quantization to models using a representative dataset. This process maps floating-point operations and weights to 8-bit integers, which reduces the model size and computational requirements while maintaining accuracy.

#### Neural network architecture deployment

The *EdgeVolution* framework relies on the Zephyr RTOS to efficiently manage microcontroller resources. Zephyr is an open-source, highly configurable framework designed specifically for embedded devices. Its robust support for hardware abstraction enables *EdgeVolution* to target a wide range of microcontrollers, promoting scalability and supporting the democratization of Edge AI across diverse applications. Additionally, Zephyr provides the runtime environment necessary for executing TFLite Micro models and includes additional built-in features critical for embedded AI deployment, such as efficient power management, multithreading, and peripheral communication. The converted and quantized TFLite Micro models are integrated into Zephyr applications by compiling them as C arrays.

#### Inference time measurement

Inference time measurements are conducted by interfacing with the microcontroller via a USB CDC ACM, commonly referred to as a virtual COM port. After flashing the microcontroller with the neural network architecture, it waits for a start command from the host computer. This command, represented by the character ’s’ (for “start"), is sent after initiating the power consumption measurement. Upon receiving the start command, the microcontroller records its current uptime to mark the beginning of the inference measurement.

The neural network inference process then begins, and upon completion, the microcontroller records its uptime again. The inference time is calculated by subtracting the recorded start time from the end time.

### Application examples

To demonstrate the broad applicability and robustness of *EdgeVolution*, we selected four diverse datasets spanning different domains. Each dataset represents longitudinal data and reflects a specific type of real-world data processing and classification task commonly performed on embedded devices at the application level. In the following sections, we present each dataset, its modality, and the corresponding classification task it addresses.

#### Cifar-10

Image classification is one of the most common tasks in intelligent systems. To evaluate the capability of our pipeline for optimizing neural networks in image-based classification, we utilized the CIFAR-10 dataset^49^. This dataset consists of 32 × 32 color images categorized into ten mutually exclusive classes: *airplane*, *automobile*, *bird*, *cat*, *deer*, *dog*, *frog*, *horse*, *ship*, and *truck*. The dataset is comprising 50,000 training images and 10,000 test images. For our optimization process, we partitioned the official 50,000 training samples into 90% for model weight training and 10% for validation. The evolutionary algorithm optimizes the validation accuracy derived from this 10% split. The official 10,000-sample test set was reserved exclusively for the final performance evaluation of the optimized models. The input images were normalized to a range of [0, 1].

#### Speech commands

We selected the Speech Commands dataset^50^ to represent data from the audio domain. This dataset consists of spoken keywords commonly used in speech processing pipelines, such as wakeword detection. Each audio file has a maximum duration of one second and contains a single spoken word. The dataset includes the following classes: *yes*, *no*, *up*, *down*, *left*, *right*, *on*, *off*, *stop*, and *go*. To create a 12-class classification problem, two additional categories were introduced: *unknown words* and *silence*.

During preprocessing, the audio data was downsampled from 16 kHz to 6 kHz. This reduction preserved the accuracy of baseline models while improving inference time and energy efficiency^24^. Additionally, the reduced sampling rate lowered peak RAM usage, enabling the deployment of wider neural network architectures without exceeding memory constraints. The default training/validation/testing split of the dataset was used.

#### sEMG dataset

Another application of Edge AI is the processing and analysis of sEMG signals, a typical closed-loop use case^51^. Real-world sEMG applications require both high accuracy and real-time data processing to ensure reliable performance. One of the use cases is the myocontrol of prostheses with multiple active DoFs for people with congenital limb differences or surgical amputations. To evaluate the capability of *EdgeVolution* in meeting these demands, we utilized a previously published sEMG dataset^52,53^. This dataset was collected using two Myo armbands, each with eight channels, to record sEMG signals from the right arm of healthy participants. Recordings were obtained from six participants performing seven distinct hand and wrist gestures, which serve as the classes for our classification task: *rest*, *hand opening*, *hand closing*, *wrist extension*, *wrist flexion*, *wrist pronation*, and *wrist supination*. In this study, participants utilized their sEMG signals to control a prosthesis with three active DoFs. As the study consisted of multiple sessions per participant, the dataset includes donning and doffing of the sensors to an approximate physiological landmark, and therefore, an increased data variability.

A sliding window of size 20 data points with a step size of 1 was applied to the data, corresponding to durations of 100 ms and 5 ms, respectively, based on the sampling frequency of 200 Hz. Our preprocessing pipeline employed only standard Notch and Band-Pass filtering, as we hypothesized that neural networks would naturally learn to filter remaining noise during training. The input data were normalized to the range [−1, 1], and the channels of each window were stacked to form an input shape of (16, 20, 1), which serves as the input for the downstream classification task. Data from the six participants was randomly partitioned once into training (three participants), validation (two participants), and testing (one participant) sets. This single partitioning was maintained consistently across all experiments.

#### DaLiAc

The DaLiAc dataset^54^ comprises IMU (inertial measurement unit) data collected from 19 participants, with sensor nodes placed at various body locations: wrist, hip, chest, and ankle. For the *EdgeVolution* experiments, only the accelerometer data from the IMU at the wrist was used, simulating a setup similar to a smartwatch accelerometer. Based on these signals, the following activity classes were formed as described in the original publication: *house* (vacuuming and sweeping), *rest* (sitting, lying, and standing), *walk* (walking, running, ascending stairs, and descending stairs), *bicycle* (cycling at 50 W and 100 W), *rope jumping*, and *washing dishes*.

To prepare the data, a sliding window of size 1024 data points (corresponding to 5 s of data, recorded at a sampling frequency of 204.8 Hz) was applied. The window was shifted across the entire dataset for each participant with a 50% overlap to ensure adequate coverage and variability. For preprocessing, the magnitude of the accelerometer data was computed from the X, Y, and Z axes, yielding a single-channel signal. The magnitude was then normalized by subtracting its mean and dividing by its standard deviation (z-score). Data from the 19 participants was randomly partitioned once into training (14 participants), validation (three participants), and testing (two participants) sets. This single partitioning was maintained consistently across all experiments.

### Hyperparameters

Although *EdgeVolution* provides an end-to-end pipeline for optimizing neural network architectures, it still requires the specification of certain hyperparameters. These hyperparameters depend on factors such as the dataset, the available hardware used for optimization, and the specific requirements of the final neural network architecture and its application. To streamline and manage these configurations, all hyperparameters are organized using the Hydra library for Python^55^. In the supplementary material, we provide examples for the hyperparameter setup for each application example (see Supplementary Listing S1).

#### Search space parameters

The design of the search space depends primarily on two key factors. First, the search space must align with the operations supported by the Edge AI framework used for inference, which in our case is TFLite Micro. Second, it must be tailored to the specific requirements of the machine learning problem being addressed.

In this study, we utilized a search space consisting of 2D convolutional (Conv) layers as part of the main building blocks (see Supplementary Listing S2/S3 and Fig. S8), similar to the search space used by Liashchynskyi et al.^56^. According to White et al.^40^, our search space is a combination of chain-structured and cell-based search spaces. To ensure compatibility with this approach, data was prepared and preprocessed to enable the application of 2D Conv layers. For single-channel longitudinal data, such as audio or accelerometer data (e.g., XYZ magnitude), we transformed the data from the time domain to the time-frequency domain using the STFT. This preprocessing step was integrated into the search space, making it an intrinsic part of the neural architecture and the end-to-end optimization pipeline. This approach addresses several key challenges: (a) it accounts for potential RAM usage peaks caused by specific STFT parameter choices, ensuring these remain within hardware constraints; (b) it facilitates the joint optimization of preprocessing and neural network architecture; and (c) it enables direct deployment of the final neural network on the microcontroller without requiring an additional digital signal processing pipeline. For implementing the STFT and associated layers, such as mel-spectrogram scaling, we used the kapre library^57^. For image data or multi-channel longitudinal data, the 2D Conv search space was utilized without time-frequency preprocessing.

#### Neural network training

Neural network training impacts the performance evaluation of each candidate neural architecture. Selecting the training parameters involves a trade-off between the runtime of the entire pipeline, the expressiveness of the results, and computational costs, particularly in terms of energy consumption during training. The most critical parameters in this context include the number of training epochs (n_epochs_cifar10_ = 20, n_epochs_speech_commands_ = 3, n_epochs_emg_dataset_ = 50, n_epochs_daliac_ = 30), the initial learning rate (lr = 0.001), the use of a learning rate decay function (exponential decay with decay_steps = 5 and decay_rate = 0.8), the extent to which the learning rate is reduced over time, and the choice of optimizer (Adam) for backpropagating the neural network weights.

#### Evolutionary optimization parameters

In the context of evolutionary optimization, we have designed a set of hyperparameters that are intuitive and easy to configure, lowering the barrier for users in their selection and application. The primary hyperparameters include the number of evolutionary generations (*n*_generations_ = 30), population size (*n*_initial_population_size_ = 500), the number of models considered in the selection process (*n*_initial_selection_size_ = 100), mutation rate (*n*_initial_mutation_rate_ = 30%), and the maximum number of feature and classification layers to constrain the search space (for Cifar-10 and sEMG datasets: *n*_feature_layers_ = 20, *n*_classification_layers_ = 10; for Speech Commands and DaLiAc datasets: *n*_feature_layers_ = 8, *n*_classification_layers_ = 4). Furthermore, we use dynamic parameter decay, adjusting values such as population size, the number of models for selection, and mutation rate over time/generations to refine the optimization process. The values used there can be seen in Supplementary Fig. S9. Modifying these hyperparameters involves a trade-off between a more thorough exploration of the search space and the associated computational costs.

#### Microcontroller boards and power measurement

Selecting the microcontroller to be used also involves determining two important parameters. The first is the allowable amount of RAM allocated to the neural network for inference, which acts as a hard (implicit) constraint. The second is dependent on the idle power consumption of the selected microcontroller, which varies between devices. To reliably detect inference cycles, a threshold for power consumption must be defined as a hyperparameter, which can be done by utilizing a script that is provided. In our study, we assume that the entire amount of RAM available on a microcontroller can be utilized for neural network inference.

#### Fitness function

In our study, the fitness function *F* is defined as a weighted sum of three objectives: accuracy (*A*), ROM usage (*M*), and energy consumption (*E*):

1 $$F={w}_{{{\rm{A}}}}\cdot A+{w}_{{{\rm{M}}}}\cdot {M}_{{{\rm{norm}}}}+{w}_{{{\rm{W}}}}\cdot {E}_{{{\rm{norm}}}},$$

where *w*_A_, *w*_M_, and *w*_E_ are the respective weights for each objective. The value of *F* is constrained to the interval [0, 1] to ensure comparability and stability across objectives.

Accuracy (*A*) is inherently bounded between 0 and 1 and is scaled directly by its weight *w*_A_. ROM usage (*M*) and energy consumption (*E*) are normalized using predefined lower bounds (hyperparameters) to ensure that their contributions to the fitness function are also within the interval [0, 1]. The normalized values *M*_norm_ and *E*_norm_ are computed as:

$${M}_{{{\rm{norm}}}}=\min \left(1,\frac{{M}_{\min }}{M}\right),\,\,{E}_{{{\rm{norm}}}}=\min \left(1,\frac{{E}_{\min }}{E}\right),$$

where ${M}_{\min }$ and ${E}_{\min }$ are the minimum possible ROM usage and energy consumption defined, respectively. During optimization, achieving values closer to these minima increases the fitness score. However, values below these thresholds are capped at a maximum contribution of 1 to maintain consistency. This fitness formulation ensures that the fitness function rewards improvements in accuracy, ROM efficiency, and energy consumption while balancing the trade-offs between these objectives according to the specified weights.

### Statistics and reproducibility

All experiments were performed three times unless otherwise specified. The Shapiro–Wilk test was used to assess the normality of data distributions. When data did not follow a normal distribution, we used the Mann–Whitney *U* test for group comparisons, particularly when evaluating performance differences between neural network architectures with and without Mel-scaling. Statistical significance was set at *p* < 0.05, with additional notation to indicate different significance thresholds (e.g., *p* < 0.05: *, *p* < 0.01: **, *p* < 0.001: ***). Bonferroni correction was applied for multiple comparisons. Throughout the results, we represent data variability using the 25th to 75th percentiles, shown as shaded areas in figures displaying performance metrics across evolutionary generations. For all statistical analyses, exact *p* values are reported for both significant and non-significant results (either in the main manuscript or the supplementary notes).

## Supplementary information

Transparent Peer Review file

Supplementary Information

## Data Availability

All of the datasets used are either publicly available or can be made available upon request.

## Code availability

*EdgeVolution* is available as a GitHub repository, which can be accessed via our GitHub page https://github.com/ankilab/EdgeVolution. This resource is fully open-source and provides users with documentation (https://ankilab.github.io/EdgeVolution/) and the use cases used in this study as a starting point to use the project for their own machine learning tasks. In addition, we use Discord to host a forum for users to get support from the community, in addition to traditional ways (email or GitHub).
