# Supplementary material for: EdgeVolution: democratizing multi-objective neural architecture search and end-to-end deployment on microcontrollers
Source: Commun Eng. 2026 Jun 23;5:113. doi: 10.1038/s44172-026-00708-2 (PMC13291272; doi:10.1038/s44172-026-00708-2)
Supplement: Supplementary file 2 — Supplementary Information [file 44172_2026_708_MOESM2_ESM.pdf]

Supplementary Notes:

EdgeVolution: Democratizing Multi-Objective Neural Architecture Search and End-to-End Deployment on Microcontrollers

René Groh[1*], Stefan Dendorfer[1], Mateo Avila Pava[1], Fabio Egle[1], Sebastian Zimmermann[1], Andreas M. Kist[1]

[1]Department Artificial Intelligence in Biomedical Engineering, Friedrich-Alexander-Universität Erlangen-Nürnberg, Nürnberger Straße 74, Erlangen, 91052, Bavaria, Germany.

*Corresponding author(s). E-mail(s): rene.groh@fau.de;

Supplementary Note 1 Failure of proxy metrics

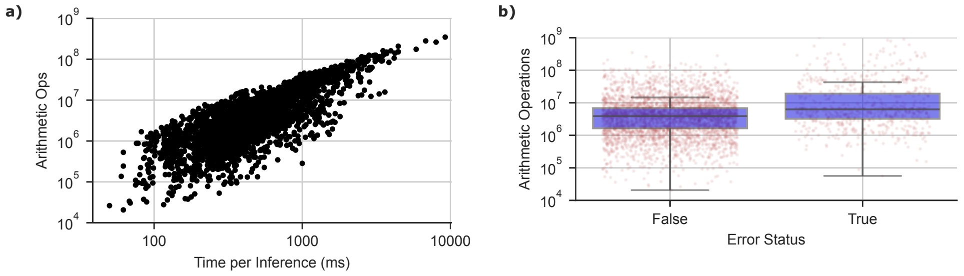

Fig. S1 Inadequacy of Operation Count as a Proxy Metric on Microcontrollers. a) Scatter plot comparing the theoretical number of arithmetic operations (a common NAS proxy metric) against the actual measured inference time on the nRF52840 microcontroller. The spread for any given operation count illustrates that theoretical complexity is a poor predictor of real-world latency on constrained hardware. b) Distribution of arithmetic operations for successful deployments (Error Status: False) versus failed deployments (Error Status: True, often due to RAM overflow). The overlap between the two groups demonstrates that proxy metrics cannot reliably predict whether a neural architecture will fit within the strict memory constraints of a microcontroller.

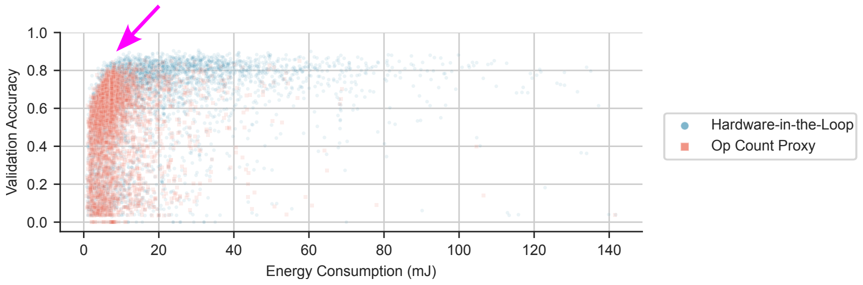

Fig. S2 Ablation Study: Hardware-in-the-Loop vs. Proxy-based Optimization. Comparison of the final populations from two distinct optimization strategies: one utilizing full hardware-in-the-loop evaluation (blue points) and one relying solely on Operation Count as a proxy for fitness (red points). The objective in both runs was to maximize accuracy ($w_A = 0.7$) while minimizing resource cost ($w_{cost} = 0.3$). The proxy-based search collapses toward small, low-operation models, failing to explore the full capabilities of the hardware. In contrast, the hardware-in-the-loop approach successfully identifies the region indicated by the magenta arrow, representing the optimal Pareto front where higher energy consumption yields gains in accuracy. The proxy approach ignores deployment constraints (e.g., RAM usage), whereas HITL naturally filters out non-deployable architectures, resulting in a more robust and hardware-efficient population.

Supplementary Note 2 Search Strategy Comparison

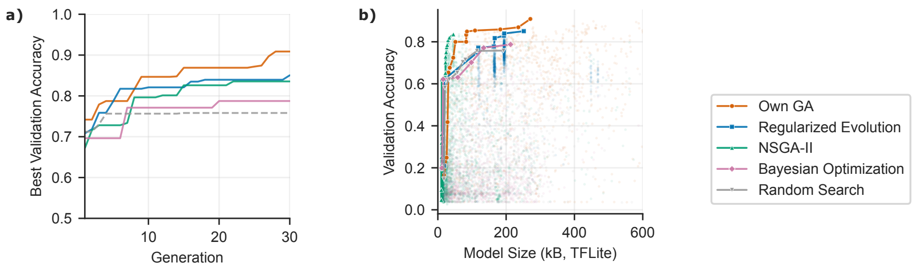

Fig. S3 Comparison of five search strategies on the Speech Commands dataset (nRF52840 target, 30 generations, accuracy-only objective). (a) Best validation accuracy over evolutionary generations. The custom genetic algorithm (Own GA) achieves the highest final accuracy (0.909), followed by Regularized Evolution (0.850), NSGA-II (0.835), Bayesian Optimization (0.787), and Random Search (0.758). (b) Explored architecture space showing validation accuracy versus TFLite model size. Each dot represents an individual architecture evaluated during the search; solid lines with markers trace the upper envelope of each strategy's explored space. The Own GA discovers higher-accuracy architectures across a wider range of model sizes compared to the other strategies.

Supplementary Note 3 Search time analysis

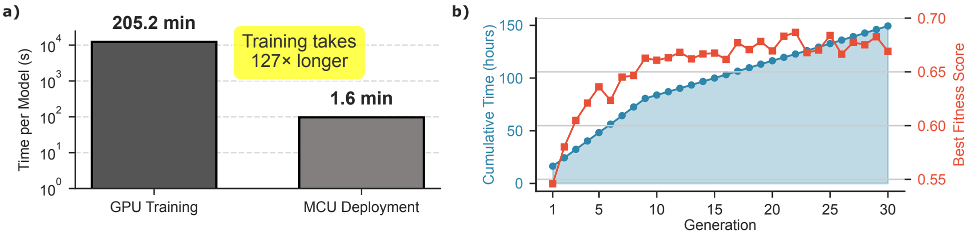

Fig. S4 Search Time Analysis and Computational Cost. Analysis of a complete evolutionary optimization run on the Speech Commands dataset targeting the nRF52840 microcontroller (Host setup: Intel Xeon W-1390P CPU, NVIDIA RTX A5000 GPU). a) Comparison of average wall-clock time per model for GPU training versus on-device deployment and measurement. The hardware-in-the-loop evaluation is approximately 127× faster than the training process, demonstrating that physical deployment is not the primary computational bottleneck. b) Cumulative search time over 30 generations plotted against the best fitness score trajectory. The total optimization time was 149.4 hours (approx. 6.2 days), during which 4,600 neural network architectures were fully trained and evaluated. Notably, GPU training accounts for 99.0% of the total runtime, confirming that the cost of hardware-in-the-loop integration is negligible in terms of search time compared to model training.

Supplementary Note 4 Surrogate Evaluation

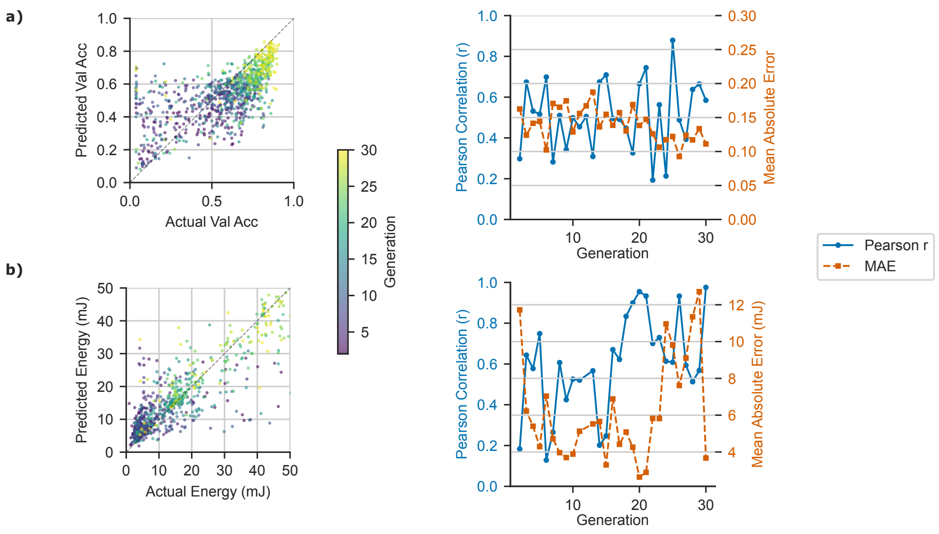

Fig. S5 Evaluation of surrogate models for predicting validation accuracy and hardware energy consumption. (a) Predicted versus actual validation accuracy for a Random Forest surrogate (100 estimators) trained on generation 1 architecture encodings (N=100) and evaluated in non-skipping mode across all 30 generations (Pearson r = 0.65, MAE = 0.14, N = 946). Points are colored by generation. (b) Predicted versus actual energy consumption (mJ) for a Random Forest surrogate trained incrementally on measured energy values from the nRF52840 target (Pearson r = 0.72, MAE = 6.0 mJ, N = 770). Right panels show the per-generation Pearson correlation (blue) and mean absolute error (orange) for each surrogate. The energy surrogate's correlation improves in later generations, reaching r > 0.95 in generations 20 and 30.

Supplementary Note 5 Hardware Lookup Table Evaluation

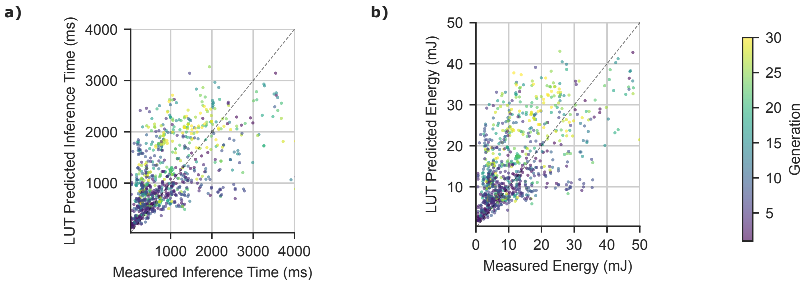

Fig. S6 Evaluation of the hardware lookup table (LUT) for predicting hardware metrics on the nRF52840 microcontroller. The LUT was built by training Random Forest models (100 estimators) on architecture encodings paired with measured hardware metrics from an initial profiling run (N = 1,000 architectures). A full 30-generation search was then performed using LUT predictions while simultaneously collecting actual on-device measurements for validation. (a) LUT-predicted versus measured inference time (Pearson r = 0.60, N = 943). (b) LUT-predicted versus measured energy consumption (Pearson r = 0.59, N = 943). Points are colored by generation. Dashed lines indicate perfect prediction.

Supplementary Note 6 EvoVis Dashboard

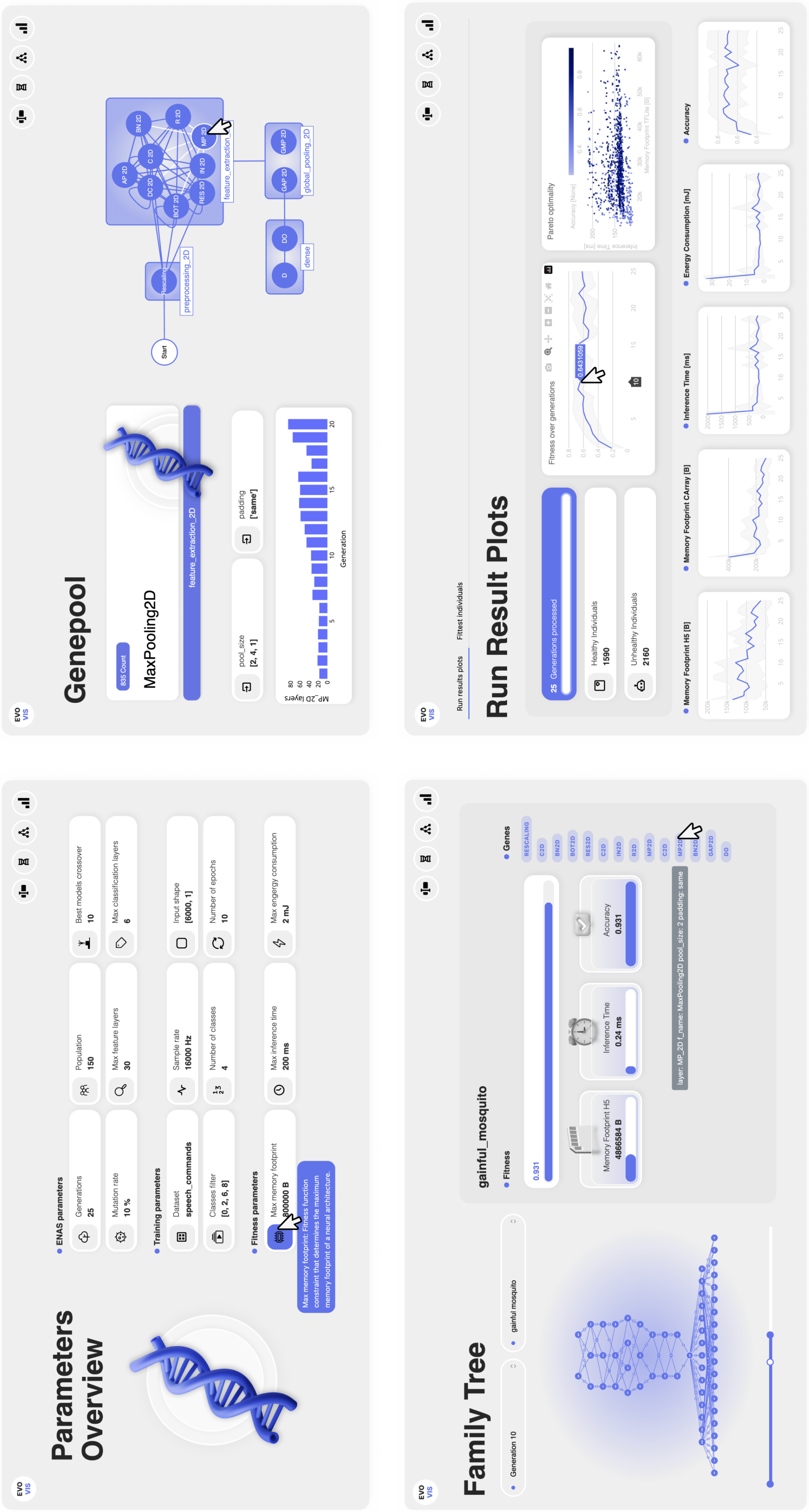

Fig. S7 EvoVis dashboard overview of the different pages showing hyperparameters, search space, family tree and NAS results.

Supplementary Note 7 NAS Hyperparameters

Listing S1 Example (Speech Commands dataset) for the hyperparameters used in *EdgeVolution*. This file must be defined for each dataset in order to be able to perform the optimization. In the future, it is conceivable that this will be abstracted to a graphical user interface.

```yaml
### General parameters:
min_free_space_gpu:  # Minimum free space in GPU in bytes to start a new training (as we train in
↪  parallel)
  value: 6_000_000
limit_tensor_arena_size: # Limit the tensor arena size in bytes (if None, it will use the maximum
↪  available tensor arena size)
  value: null

### Dataset/DNN training hyper-parameters:
results_path:
  value: "Results/"
dataset_name:
  value: "speech_commands"
sample_rate:
  value: 6000
input_shape:
  value: [6000, 1]
num_classes:
  value: 12
top_activation:
  value: "softmax"
num_epochs:
  value: 3
batch_size:
  value: 128
optimizer:
  value: "adam"
loss:
  value: "categorical_crossentropy"
metrics:
  value: ["accuracy"]

### EdgeVolution hyper-parameters:
num_generations:
  value: 30
population_size_decay:
  value: [[1, 5], [2, 250], [10, 100]] # [(from_generation, population_size), (from_generation,
  ↪  population_size), ...]
num_best_models_crossover_decay:
  value: [[1, 100], [2, 50], [10, 20]] # [(from_generation, population_size), (from_generation,
  ↪  population_size), ...]
mutation_rate_decay:
  value: [[1, 30], [2, 25], [5, 20], [10, 15]] # [(from_generation, population_size),
  ↪  (from_generation, population_size), ...]
max_num_feature_layers:
  value: 8
max_num_classification_layers:
  value: 4

### Fitness
min_rom_usage:
  value: 150_000
min_energy_information:
  value: 0.5
acc_weight:
  value: 0.7
rom_usage_weight:
  value: 0.1
energy_information_weight:
  value: 0.2
```

Supplementary Note 8 Search space

Listing S2 Search space (gene pool) configuration for neural architecture search. Values are following either the scheme ["option1", "option2", "option3"] or [start, stop, step].

```
gene_pool:
  preprocessing_2D:
    - layer: STFT_2D
      f_name: STFT
      n_fft: [64, 512, 16]
      hop_length: [128, 396, 16]
      input_data_format: ['channels_last']
      output_data_format: ['channels_last']

    - layer: MAG_2D
      f_name: Magnitude()

    - layer: FB_2D
      f_name: get_filterbank_layer
      type: ['mel']
      n_mels: [32, 128, 8]
      mel_f_min: [0, 0, 1]
      mel_f_max: [3000, 3000, 1]
      output_data_format: ['channels_last']

    - layer: MAG2DEC_2D
      f_name: MagnitudeToDecibel()

  feature_extraction_2D:
    - layer: C_2D_BLOCK
      f_name: get_conv2d_block
      filters: [4, 64, 1]
      kernel_height: [1, 12, 1]
      kernel_width: [1, 12, 1]
      strides: [1, 2, 1]
      padding: ['same']
      norm_layer: ['None', 'BatchNormalization']
      activation: ['None', 'relu', 'sigmoid', 'tanh', 'leaky_relu']

    - layer: DC_2D_BLOCK
      f_name: get_depthwise_conv2d_block
      kernel_height: [1, 12, 1]
      kernel_width: [1, 12, 1]
      strides: [1, 2, 1]
      padding: ['same']
      norm_layer: ['None', 'BatchNormalization']
      activation: ['None', 'relu', 'sigmoid', 'tanh', 'leaky_relu']

    - layer: C_2D
      f_name: Conv2D
      filters: [4, 48, 1]
      kernel_size: [1, 5, 1]
      strides: [1, 2, 1]
      padding: ['same']
      activation: ['relu']

    - layer: DC_2D
      f_name: DepthwiseConv2D
      kernel_size: [1, 5, 1]
      strides: [1, 2, 1]
      padding: ['same']
      activation: ['relu']

    - layer: MP_2D
      f_name: MaxPooling2D
      pool_size: [2, 4, 1]
      padding: ['same']

    - layer: AP_2D
      f_name: AveragePooling2D
      pool_size: [2, 4, 1]
      padding: ['same']

    - layer: BN_2D
      f_name: BatchNormalization()

  global_pooling_2D:
    - layer: GAP_2D
      f_name: GlobalAveragePooling2D()

    - layer: GMP_2D
      f_name: GlobalMaxPooling2D()

  dense:
    - layer: D
      f_name: Dense
      units: [8, 64, 8]
      activation: ['relu']
```

Listing S3 Rule set that defines how each block of the defined search space is connected with each other.

```
rule_set:
  ###################################################
  # Sorted by groups. Each group is a list of layers, where 'rule' is a list of layers that can
  ↪  be used after the current layer.
  ###################################################

  Start:
    rule: ["STFT_2D"]

  ###################################################
  # 2D layers
  ###################################################

  STFT_2D:
    rule: ["MAG_2D"]

  MAG_2D:
    rule: ["C_2D_BLOCK", "DC_2D_BLOCK", "FB_2D", "MAG2DEC_2D"]

  FB_2D:
    rule: ["C_2D_BLOCK", "DC_2D_BLOCK", "MAG2DEC_2D"]

  MAG2DEC_2D:
    rule: ["C_2D_BLOCK", "DC_2D_BLOCK"]

  C_2D_BLOCK:
    rule: ["AP_2D", "MP_2D", "C_2D_BLOCK", "DC_2D_BLOCK"]

  DC_2D_BLOCK:
    rule: ["AP_2D", "MP_2D", "C_2D_BLOCK", "DC_2D_BLOCK"]

  MP_2D:
    rule: ["C_2D_BLOCK", "DC_2D_BLOCK"]

  AP_2D:
    rule: ["C_2D_BLOCK", "DC_2D_BLOCK"]

  GAP_2D:
    rule: ["D"]

  GMP_2D:
    rule: ["D"]

  ###################################################
  # Dense layers
  ###################################################

  D:
    rule: ["D"]

###################################################
# Rule set group just for Dashboard visualization
###################################################
rule_set_group:
  - group: "feature_extraction_2D"
    rule: ["global_pooling_2D"]
```

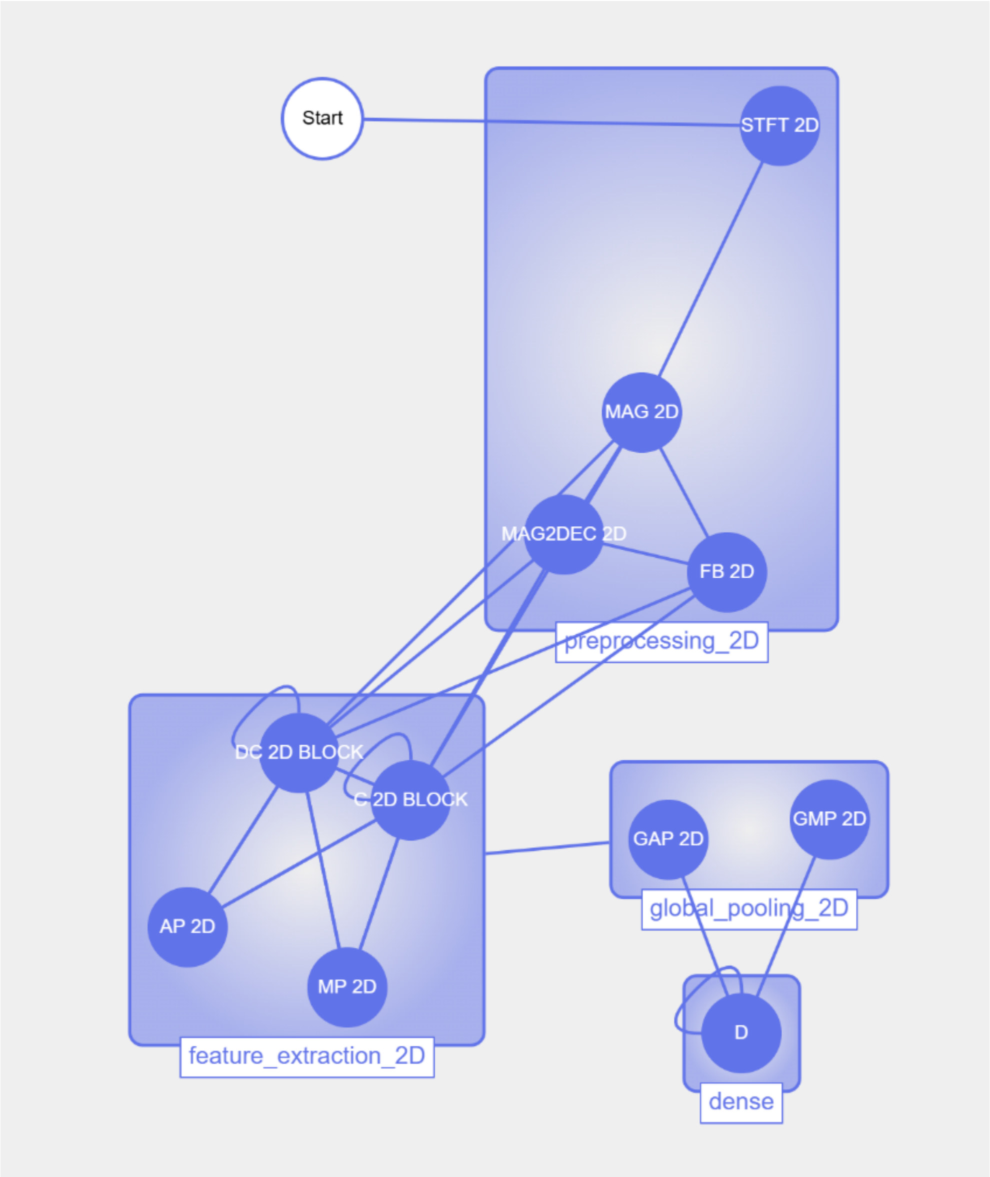

Fig. S8 Search space visualization of the EvoVis dashboard where the search space and the rule set are both used.

Supplementary Note 9 Available operations in TFLite Micro/LiteRT

Table S1: List of TensorFlow Lite Micro operations with indication if the respective kernel is available in CMSIS-NN, which offers faster implementations.

Operation	Description	CMSIS-NN
Abs	Computes the absolute value of input tensor elements	×
Add	Element-wise addition of two tensors	✓
AddN	Adds all input tensors element-wise	×
ArgMax	Returns the indices of the maximum values along an axis	×
ArgMin	Returns the indices of the minimum values along an axis	×
AssignVariable	Assigns a value to a variable	×
AveragePool2D	Performs average pooling on the input	✓
BatchMatMul	Performs batch matrix multiplication	✓
BatchToSpaceNd	Reshapes batch dimension into spatial dimensions	×
BroadcastArgs	Returns the shape of broadcasted tensors	×
BroadcastTo	Broadcasts an array to a compatible shape	×
CallOnce	Calls a function once	×
Cast	Casts a tensor to a new type	×
Ceil	Computes ceiling of elements in the input tensor	×
CircularBuffer	Implements a circular buffer data structure	×
Concatenation	Concatenates tensors along one dimension	×
Conv2D	Performs 2D convolution	✓
Cos	Computes cosine of input elements	×
CumSum	Computes the cumulative sum of elements along an axis	×
Delay	Signal processing operation that introduces time delay	×
DepthToSpace	Rearranges depth data into spatial dimensions	×
DepthwiseConv2D	Performs depthwise 2D convolution	✓
Dequantize	Converts quantized tensors back to floating-point	×
DetectionPostprocess	Post-processing for object detection models	×

Continued on next page

Table S1 – Continued from previous page

Operation	Description	CMSIS-NN
Div	Element-wise division of two tensors	×
EmbeddingLookup	Looks up embeddings for specified IDs	×
Energy	Computes signal energy in signal processing	×
Elu	Exponential Linear Unit activation function	×
Equal	Element-wise equality comparison	×
EthosU	Operations for Arm Ethos-U NPU	×
Exp	Computes exponential of input elements	×
ExpandDims	Expands tensor dimensions by inserting a dimension	×
FftAutoScale	Auto-scales Fast Fourier Transform output	×
Fill	Creates a tensor filled with a scalar value	×
FilterBank	Applies filter bank in signal processing	×
FilterBankLog	Applies logarithmic filter bank	×
FilterBankSquareRoot	Applies square root to filter bank outputs	×
FilterBankSpectralSubtraction	Applies spectral subtraction to filter bank	×
Floor	Computes floor of elements in the input tensor	×
FloorDiv	Integer division with floor operation	×
FloorMod	Returns element-wise remainder of division	×
Framer	Frames a signal into overlapping windows	×
FullyConnected	Performs fully connected layer operations	✓
Gather	Gathers slices from params tensor	×
GatherNd	Gathers slices from params tensor at indices	×
Greater	Element-wise greater than comparison	×
GreaterEqual	Element-wise greater than or equal comparison	×
HardSwish	Hard version of swish activation function	×
If	Conditional execution of subgraphs	×
Irfft	Inverse real Fast Fourier Transform	×
L2Normalization	L2 normalization of input tensor	×
L2Pool2D	L2 pooling operation	×
LeakyRelu	Leaky version of ReLU activation function	×
Less	Element-wise less than comparison	×
LessEqual	Element-wise less than or equal comparison	×
Log	Computes natural logarithm of input elements	×
LogicalAnd	Element-wise logical AND operation	×
LogicalNot	Element-wise logical NOT operation	×

Continued on next page

Table S1 – Continued from previous page

Operation	Description	CMSIS-NN
LogicalOr	Element-wise logical OR operation	×
Logistic	Computes sigmoid activation function	×
LogSoftmax	Computes log softmax activation function	×
Maximum	Element-wise maximum of two tensors	✓
MaxPool2D	Performs max pooling on the input	✓
MirrorPad	Pads a tensor using mirroring	×
Mean	Computes mean of elements across dimensions	×
Minimum	Element-wise minimum of two tensors	✓
Mul	Element-wise multiplication of two tensors	✓
Neg	Negates the value of each element	×
NotEqual	Element-wise inequality comparison	×
OverlapAdd	Reconstructs signal from overlapped frames	×
Pack	Packs a list of tensors into a single tensor	×
Pad	Pads a tensor with a constant value	✓
PadV2	Extended version of Pad with more options	×
PCAN	Per-Channel Affine Normalization for audio	×
Prelu	Parametric Rectified Linear Unit activation	×
Quantize	Quantizes a tensor from floating-point to integer	×
ReadVariable	Reads the value of a variable	×
ReduceMax	Computes maximum of elements across dimensions	×
Relu	Rectified Linear Unit activation function	×
Relu6	ReLU capped at 6 activation function	×
Reshape	Changes the shape of a tensor	×
ResizeBilinear	Resizes images using bilinear interpolation	×
ResizeNearestNeighbor	Resizes images using nearest neighbor interpolation	×
Rfft	Real Fast Fourier Transform	×
Round	Rounds values to the nearest integer	×
Rsqrt	Computes reciprocal square root	×
SelectV2	Selects elements from two tensors based on condition	×
Shape	Returns shape of a tensor	×
Sin	Computes sine of input elements	×
Slice	Extracts a slice from a tensor	×
Softmax	Computes softmax activation function	✓

Continued on next page

Table S1 – Continued from previous page

**Operation**	**Description**	**CMSIS-NN**
SpaceToBatchNd	Reshapes spatial dimensions into batch dimension	×
SpaceToDepth	Rearranges spatial data into depth dimension	×
Split	Splits a tensor into sub-tensors along a dimension	×
SplitV	Splits a tensor into sub-tensors with specified sizes	×
Squeeze	Removes dimensions of size 1 from the tensor shape	×
Sqrt	Computes square root of input elements	×
Square	Computes square of input elements	×
SquaredDifference	Computes squared difference between two tensors	×
StridedSlice	Extracts a strided slice from a tensor	×
Stacker	Stacks frames for signal processing	×
Sub	Element-wise subtraction of two tensors	×
Sum	Computes sum of elements across dimensions	×
Svdf	Singular Value Decomposition Filter operation	✓
Tanh	Computes hyperbolic tangent of input elements	×
TransposeConv	Performs transposed 2D convolution	✓
Transpose	Permutes dimensions of a tensor	✓
Unpack	Unpacks a tensor into a list of tensors	×
UnidirectionalSequenceLSTM	Applies LSTM to an input sequence	✓
VarHandle	Creates a resource variable handle	×
While	Executes a subgraph repeatedly	×
Window	Applies window function to signal frames	×
ZerosLike	Creates a tensor of zeros with same shape as input	×

Supplementary Note 10 Visualization of optimization decays

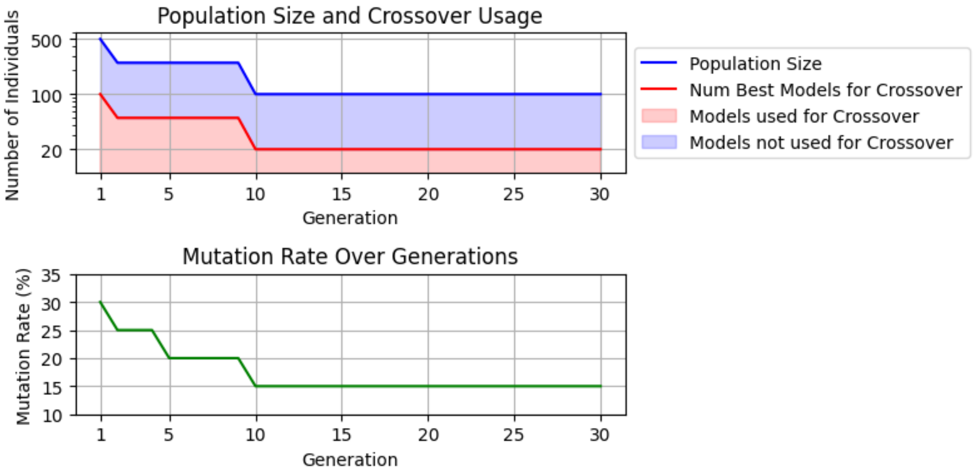

Fig. S9 Visualization of the decays used for population size and mutation rate. These are hyperparameter that are specified in the config file as well.

Supplementary Note 11 Power consumption measurement

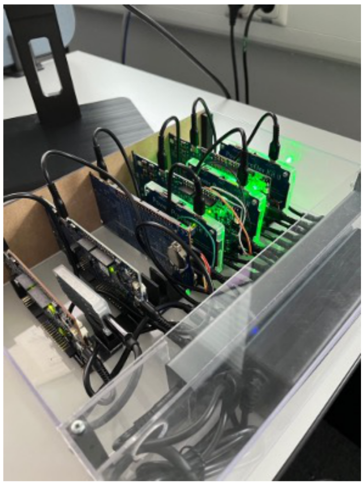

Fig. S10 Image of the power measurement setup using the three Nordic development kits (nRF52833, nRF52840, nRF5340).

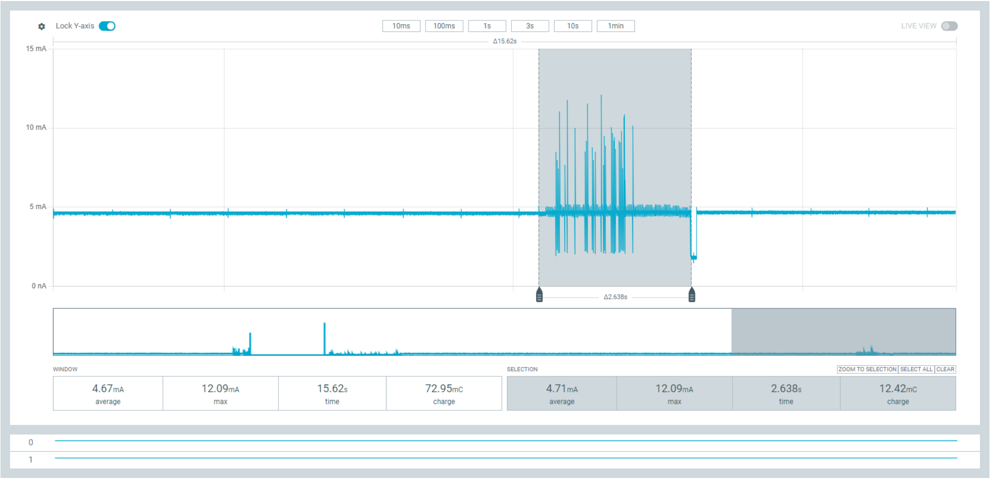

Fig. S11 Visualization of the current consumption measurement. The microcontroller is in an active/idle mode with a baseline current consumption visually around 4.6 mA. During inference (selected period), the average current consumption is 4.71 mA over 2.638 s, resulting in a measured charge of 12.42 mC. This data is then used to calculate the energy consumption with the supply voltage of 3.3 V. Directly after inference ends, the microcontroller is set to sleep for 100 ms (indicated by the sharp drop in current).

Supplementary Note 12 Statistical Testing of Mel-Scaling usage

Table S2 Shapiro-Wilk Test Results for Normality and Statistical Significance of Validation Accuracy

**Normality (No n_mels)**	**Normality (Has n_mels)**	**Significance (Val_Acc)**
	**Worst 25%**	
False ($p = 3.43 \times 10^{-37}$)	False ($p = 4.71 \times 10^{-22}$)	True ($p = 1.12 \times 10^{-23}$)
	**Middle 50%**	
False ($p = 5.68 \times 10^{-41}$)	False ($p = 2.14 \times 10^{-8}$)	True ($p = 2.24 \times 10^{-7}$)
	**Best 25%**	
False ($p = 2.72 \times 10^{-29}$)	True ($p = 0.216$)	True ($p = 0.00179$)

Supplementary Note 13 Expansion by other hardware targets: STM32H753ZI as an example

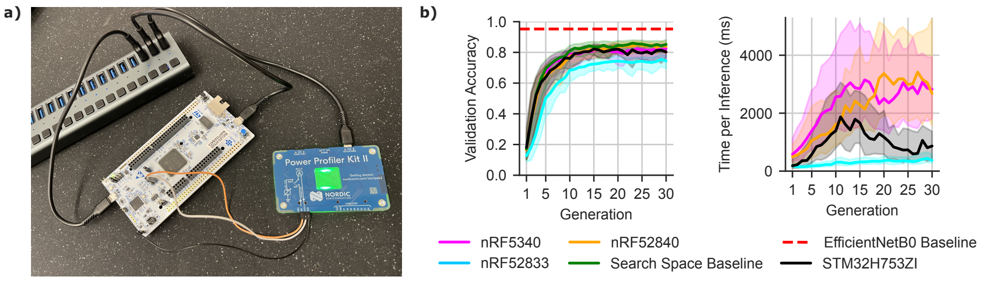

Fig. S12 *EdgeVolution* with STM32H753ZI as hardware target (480 MHz Arm Cortex-M7 processor with 2 MB of Flash memory and 1 MB of RAM). For power consumption measurements the Nordic Power Profiler Kit 2 and Zephyr RTOS are used. a) Image of the hardware setup. Instead of a Nordic development board, an STM Nucleo development board is used. b) The results of one exemplary run on the Speech Commands dataset is provided. Median validation accuracy and measured time per inference across evolutionary generations, with the shaded area representing the 25th to 75th percentiles. The search space baseline follows the defined search space (defined in the main manuscript) but excludes microcontroller constraints.
