# Supplementary material for: EdgeVolution: democratizing multi-objective neural architecture search and end-to-end deployment on microcontrollers
Source: Commun Eng. 2026 Jun 23;5:113. doi: 10.1038/s44172-026-00708-2 (PMC13291272; doi:10.1038/s44172-026-00708-2)
Supplement: Supplementary file 1 — Transparent Peer Review file [file 44172_2026_708_MOESM1_ESM.pdf]

nature portfolio

Peer Review File

EdgeVolution: Democratizing Multi-Objective Neural Architecture Search and End-to-End Deployment on Microcontrollers

Corresponding Author: Mr René Groh

This file contains all reviewer reports in order by version, followed by all author rebuttals in order by version.

Version 0:

Reviewer comments:

Reviewer #1

(Remarks to the Author)
The paper introduces EdgeVolution, a hardware-in-the-loop (HIL) neural architecture search (NAS) framework tailored for microcontrollers. The framework integrates Zephyr RTOS, TensorFlow Lite Micro (with CMSIS-NN kernels), and the Nordic Power Profiler Kit 2 to continuously deploy candidate models during the search and collect real latency and power consumption measurements in-loop. The system is presented as an end-to-end, open pipeline, and its evaluation spans several tasks (image classification, speech, and biomedical signals) across three Nordic Semiconductor microcontrollers. The paper also contributes a multi-objective fitness functon(balancing accuracy, ROM footprint, and energy per inference) and an interactive dashboard ("EvoVis") for monitoring the search process.

Strengths:
- The system is well-engineered and demonstrates a clean integration of software and hardware components.

- The inclusion of real power and latency measurements in the NAS loop, rather than proxy metrics, is valuable from a tooling and reproducibility standpoint.

- Demonstrating the pipeline across multiple application domains (vision, speech, biomedical) shows its practical utility.

Concerns and points for revision:
- While the paper presents an impressive system contribution, the research/methodological novelty is less clear. Hardware-in-the-loop for NAS has been demonstrated before in the context of mobile SoCs (e.g., MnasNet) and, while less common on MCUs, the approach itself is not fundamentally new. In fact, much of the recent literature has moved toward avoiding HIL evaluation due to its prohibitive time cost, favoring lookup tables, predictors, or proxy metrics instead. The current work would benefit from a clearer articulation of where it goes beyond "engineering integration" and how it advances research methodology. Also a complete search time analysis and number of hardware measurements is needed.

- The main limitation of HIL NAS—the time required to flash and evaluate each candidate—remains largely unaddressed. The paper briefly mentions the potential for zero- or one-shot NAS integration as future work, but provides no experimental evidence. Demonstrating at least preliminary results (e.g., a hybrid strategy where only top-k candidates are evaluated on hardware) would significantly strengthen the claim of advancing the state of the art.

- The evaluation is restricted to Nordic Semiconductor devices. While these are relevant MCU targets, the claim of a flexible, general framework would be much stronger if demonstrated on at least one additional vendor's platform (e.g., STM32, NXP, or RP2040). This would substantiate the portability claims of the Zephyr + TFLM integration.

- The paper would benefit from an ablation or comparison to proxy approaches (e.g., latency LUTs, op-count approximations) to quantify the gap between estimated vs. real measurements, as well as the trade-off in search cost. This would directly address the reviewer's concern about whether full HIL NAS is truly worth the time cost in practice. How much gain do wehave over conventional NAS such as MCUNet?

Writing comments:
- The overall paper is clearly written and well-organized. The descriptions of the pipeline components (deployment, measurement, and fitness function) are easy to follow.

- Section 2 (related work) could be tightened to emphasize the key distinction: many prior works have avoided HIL due to cost, whereas this paper embraces it, albeit without yet solving the cost barrier.

- The paper sometimes uses strong novelty claims, “first framework”, that may be overstated. It would read more convincingly if positioned as a system/tooling contribution with practical engineering novelty, rather than a fundamentally new NAS method.

Reviewer #2

(Remarks to the Author)
The paper EdgeVolution: Democratizing Multi-Objective Neural Architecture Search and End-to-End Deployment on Microcontrollers tackles the challenge of running AI on highly constrained devices like wearables, IoT nodes, and microcontrollers. While deep learning has advanced rapidly, its deployment on edge hardware is hampered by inefficiency, poor reproducibility, and the absence of hardware-specific adaptation tools.
To address this, the authors introduce EdgeVolution, a hardware-in-the-loop platform that combines neural architecture search with multi-objective optimization, balancing accuracy, latency, and energy consumption. It enables direct deployment on microcontrollers, ensuring that models are tuned to both task and hardware constraints. Demonstrations across multiple applications show its versatility.
The work is motivated by the growing gap between AI breakthroughs and their real-world implementation in constrained environments. EdgeVolution is presented as a way to close this gap, making edge AI more accessible, efficient, and reproducible.
The paper is well-written and the innovation and implementation is clear. Given this reviewer’s background in neuromorphic hardware, it seems that the work addresses an important limitation in edge-computing devices. Where neuromorphic electronics aim to embed intelligence directly in hardware for energy efficiency and adaptability, EdgeVolution does so in the digital domain by tailoring networks to the physical limits of edge devices.
One question then arrises, whether this approach can also further enhance the efficiency of hardware neural networks and neuromorphic systems applied in edge-computing and in-sensor computing devices, and how this can be envisioned in the future?

Reviewer #3

(Remarks to the Author)
The authors present an evolutionary algorithm based codesign framework called EdgeVolution for edge deployment of AI models. The paper is well written. The authors have demonstrated the framework on four different AI tasks, which is appreciated and will serve as a useful literature for the broader community.
Here are a few questions and comments that would help further improve the paper:

1. Details about the neural network model being used for each task is missing.

2. It would be useful to add details about the evolutionary optimization in EdgeVolution and impacts of mutation and cross-over fractions in your evaluation.

3. How does your evolutionary optimization method compare with other EAs such as LEAP and MENNDL? Here are the papers:
a. Steven R. Young, Derek C. Rose, Thomas P. Karnowski, Seung-Hwan Lim, and Robert M. Patton. 2015. Optimizing deep learning hyper-parameters through an evolutionary algorithm. In Proceedings of the Workshop on Machine Learning in High-Performance Computing Environments (MLHPC '15). Association for Computing Machinery, New York, NY, USA, Article 4, 1–5. https://doi.org/10.1145/2834892.2834896

b. Mark A. Coletti, Eric O. Scott, and Jeffrey K. Bassett. Library for evolutionary algorithms in python (leap). In Proceedings of the 2020 Genetic and Evolutionary Computation Conference Companion, GECCO '20, 1571–1579. New York, NY, USA, 2020. Association for Computing Machinery. URL: https://doi.org/10.1145/3377929.3398147, doi:10.1145/3377929.3398147.

4. Why does Mel scaling not perform as well or better than FFT based preprocessing in the audio application?

5. For CIFAR-10, you mention using 90% of the dataset for training and 10% for validation. Does the EA optimize for validation set? How is the test set defined?

6. In your fitness function in equation (1), what are the values for wM and wE?

7. Why does the population size decrease over generations, as shown in the supplementary document appendix E?

Version 1:

Reviewer comments:

Reviewer #1

(Remarks to the Author)
Thank you for your rebuttal.

I went through every answer one by one and I'm still missing the novelty of the work. The Zephyr real-time operating system (RTOS) for direct on-device deployment attached to the overall framework, is interesting, but it is only engineering.

Concerning the bottleneck and search time, it is true, if you include full training in the loop (which is quite impractical when you have a large search space), 99% of the end-to-end search time is dedicated to training. But this is rarely done! We usually, use a surrogate model, or approximate the ranking of the architectures, which takes a couple of ms. Then, if a surrogate model is used, the bottleneck becomes indeed the hardware in the loop evaluation.

I agree that FLOPs is definitely not a good proxy (also the case for GPUs). But one can look for a combination of features, the use of a lookup table, or the use of surrogate models. All these strategies have been proven. And the correlation figure shown in the paper appears in several papers in the literature, including Dudziak, Lukasz, et al. "Brp-nas: Prediction-based nas using gcns." Advances in neural information processing systems 33 (2020): 10480-10490. (figure 1)

I would highly suggest to include more features in your framework to achieve a descent engineering contribution to the field of HW-NAS:
- Possibility of training surrogate models for accuracy
- Possibility of building a lookup table for a given hardware target
- Possibility of training a surrogate model for a given hardware target
- Exploring multiple search strategies.

Without these, I don't see the novelty in the framework compares to what already exist in the literature and open sourced on github.

Reviewer #2

(Remarks to the Author)
The authors have addressed my comments. The manuscript can be accepted.

Reviewer #3

(Remarks to the Author)
The authors have well addressed all the comments and questions in the last review. It would be good to include the explanation for Mel scaling versus STFT in the paper as well in the Section 2.3 on Keyword spotting application. Their work demonstrating multiple applications as well as multiple MCU platforms is well appreciated, and will be a good literature contribution to the field.

Version 2:

Reviewer comments:

Reviewer #1

(Remarks to the Author)
My comments have been fully addressed.

Reviewer #3

(Remarks to the Author)
The authors have well addressed all of my comments so far. The manuscript can be accepted.

Open Access This Peer Review File is licensed under a Creative Commons Attribution 4.0 International License, which permits use, sharing, adaptation, distribution and reproduction in any medium or format, as long as you give appropriate credit to the original author(s) and the source, provide a link to the Creative Commons license, and indicate if changes were made.
In cases where reviewers are anonymous, credit should be given to 'Anonymous Referee' and the source.
The images or other third party material in this Peer Review File are included in the article's Creative Commons license, unless indicated otherwise in a credit line to the material. If material is not included in the article's Creative Commons license and your intended use is not permitted by statutory regulation or exceeds the permitted use, you will need to obtain permission directly from the copyright holder.
To view a copy of this license, visit https://creativecommons.org/licenses/by/4.0/

René Groh
Department Artificial Intelligence in Biomedical Engineering
rene.groh@fau.de

04.12.2025

The Editorial Office
Communications Engineering

Subject: Rebuttal Letter for Manuscript ID: COMMSENG-25-0589-T - "EdgeVolution: Democratizing Multi-Objective Neural Architecture Search and End-to-End Deployment on Microcontrollers"

Dear Editors,

Thank you for managing the review process for our manuscript and for providing us with the reviewers' insightful feedback. We also would like to thank the reviewers for their time and constructive comments, which have helped us to significantly improve the quality of our paper.

We have carefully considered all the points raised by the reviewers and have revised the manuscript accordingly. Below, we provide a point-by-point response to each of the reviewers' comments. All changes in the revised manuscript have been highlighted for your convenience.

Reviewer #1

We thank Reviewer #1 for their thorough evaluation and positive feedback on the engineering and practical utility of our EdgeVolution framework. We have addressed the concerns as follows:

Comment 1: "While the paper presents an impressive system contribution, the research/methodological novelty is less clear. Hardware-in-the-loop for NAS has been demonstrated before in the context of mobile SoCs (e.g., MnasNet) and, while less common on MCUs, the approach itself is not fundamentally new. In fact, much of the recent literature has moved toward avoiding HIL evaluation due to its prohibitive time cost, favoring lookup tables, predictors, or proxy metrics instead. The current work would benefit from a clearer articulation of where it goes beyond “engineering integration” and how it advances research methodology. Also a complete search time analysis and number of hardware measurements is needed."

Response: We thank the reviewer for raising this critical point regarding methodological novelty. While we acknowledge that HITL is less common in recent NAS literature due to perceived cost and technical burdens, our research demonstrates that for the specific domain of microcontrollers (MCUs), the trend toward proxies/predictors is scientifically insufficient.

MCUs impose strict, non-linear resource constraints that proxy metrics (e.g., Operation Count) cannot accurately model. We now provide an additional analysis (Appendix A, Fig. A1; lines 202-205 and 541-545) that substantiates this by showing a weak correlation between operation counts and actual inference times. Furthermore, we demonstrate that optimizing for low operation counts fails to prevent fatal errors, such as RAM overflows. Thus, HITL is not just an engineering addition, but a scientifically necessary methodology to guarantee deployability on this hardware class.

We have addressed the concern regarding prohibitive time cost by adding a search time analysis (Appendix B, lines 159-161 and lines 404-406). Our results show that GPU training accounts for 99.0% of the total runtime, while the HITL deployment and measurement take only 1.6 minutes per model (approx. 1% of total time). This confirms that HITL is not the computational bottleneck. We also clarified that our strategy successfully evaluates approximately 4,600 neural network architectures per run directly on the hardware.

Beyond immediate optimization, our exact HITL measurement pipeline captures detailed power consumption profiles. In our opinion, this granularity advances research methodology by enabling future work to analyze and optimize not just for average power, but for specific energy patterns, peak loads, or layer-wise power consumption, which is impossible with lookup tables or proxies.

Comment 2: "The main limitation of HIL NAS—the time required to flash and evaluate each candidate—remains largely unaddressed. The paper briefly mentions the potential for zero- or one-shot NAS integration as future work, but provides no experimental evidence. Demonstrating at least preliminary results (e.g., a hybrid strategy where only top-k candidates are evaluated on hardware) would significantly strengthen the claim of advancing the state of the art."

Response: We thank the reviewer for this suggestion regarding efficiency. However, our experimental data contradict the assumption that flashing/evaluation is the bottleneck.

As established in our response to Comment 1 and detailed in Appendix B, the physical deployment and measurement accounts for less than 1% of the total runtime, while training accounts for 99%. Consequently, a hybrid strategy that selectively evaluates candidates on hardware would yield negligible time savings while sacrificing ground-truth validation.

We argue that a rigid "Top-k" strategy based on accuracy is disadvantageous for microcontrollers. High-accuracy models often correlate with maximum resource usage. Selecting only the top candidates increases the probability of encountering, e.g., RAM overflows, potentially leaving the evolutionary algorithm with no valid parents. Furthermore, this approach risks filtering out smaller, highly efficient models, thereby missing the lower end of the Pareto front.

To address the reviewer’s interest in robust selection strategies (particularly for future One-Shot NAS integrations), we have added a proposal for a "Success-Driven Quota Deployment" strategy to the Discussion (lines 450-460). This approach iterates through candidates ranked by

accuracy and attempts deployment until a fixed quota (N) of successfully deployed models is reached. This prioritizes accuracy while robustly handling the high failure rate inherent to constrained hardware.

Comment 3: "The evaluation is restricted to Nordic Semiconductor devices. While these are relevant MCU targets, the claim of a flexible, general framework would be much stronger if demonstrated on at least one additional vendor's platform (e.g., STM32, NXP, or RP2040). This would substantiate the portability claims of the Zephyr + TFLM integration."

Response: We thank the reviewer for this suggestion. To demonstrate generalizability, we have expanded our evaluation to include the STM32 platform (STM32H753ZI). We added Appendix I and Figure I1, which show that the framework successfully adapts to the STM32 architecture (Cortex-M7) using the exact same pipeline. Notably, adapting the framework to this new vendor required only a simple update to the Zephyr hardware target configuration. We also mentioned this in the main manuscript in lines 399-401.

Comment 4: "The paper would benefit from an ablation or comparison to proxy approaches (e.g., latency LUTs, op-count approximations) to quantify the gap between estimated vs. real measurements, as well as the trade-off in search cost. This would directly address the reviewer's concern about whether full HIL NAS is truly worth the time cost in practice. How much gain do we have over conventional NAS such as MCUNet?"

Response: We thank the reviewer for this suggestion. To address this, we have added a dedicated Ablation Study (Appendix C) and clarified the distinction between our framework and MCUNet.

We performed a comparative run optimizing solely based on operation count versus our full HITL approach.

- Results (Figure C1): The proxy-based search failed to exploit the hardware's capacity, collapsing towards very small, low-accuracy models. It missed the high-value region of the Pareto front (indicated by the magenta arrow in Fig. C1) where higher energy usage yields accuracy gains.
- Conclusion: The "gain" of HITL is the ability to find high-performance architectures that proxies erroneously penalize or fail to identify as deployable (mentioned in lines 442-449).

While both frameworks (MCUNet and EdgeVolution) target microcontrollers, they address different design philosophies:

- MCUNet relies on co-design, optimizing the neural network specifically for its custom inference engine (TinyEngine).
- EdgeVolution optimizes for standard, widely-supported ecosystems (TFLite Micro on Zephyr RTOS). Our goal is portability and accessibility across diverse vendor hardware (as demonstrated with Nordic and STM32), rather than optimizing a specific engine-architecture pair. While TinyEngine may offer raw speed advantages, it currently

lacks the broad hardware integration of Zephyr/TFLite. Once TinyEngine is integrated into Zephyr, a direct benchmark would be a valuable direction for future research.

Writing Comments:

- "The overall paper is clearly written and well-organized. The descriptions of the pipeline components (deployment, measurement, and fitness function) are easy to follow."
  - **Response:** We thank the reviewer for that kind comment.
- "Section 2 (related work) could be tightened to emphasize the key distinction: many prior works have avoided HIL due to cost, whereas this paper embraces it, albeit without yet solving the cost barrier."
  - **Response:** We thank the reviewer for this observation. We have revised the Introduction section to clarify our position on the "cost barrier" (lines 91-94). While we acknowledge the higher computational cost of HITL, we argue that avoiding it creates a "deployability barrier." As demonstrated in our new analyses (Appendices A and C), proxy-based methods often fail to identify deployable architectures or adapt to novel hardware targets (e.g., STM32 vs. Nordic). Therefore, we accept the increased search cost not as an unsolved problem, but as a necessary trade-off to ensure practical relevance and ground-truth deployability on constrained microcontrollers.
- "The paper sometimes uses strong novelty claims, “first framework”, that may be overstated. It would read more convincingly if positioned as a system/tooling contribution with practical engineering novelty, rather than a fundamentally new NAS method."
  - **Response:** We thank the reviewer for this constructive feedback. We agree that the primary value of EdgeVolution lies in the system-level integration and practical engineering that makes HITL accessible, rather than in proposing a fundamentally new search algorithm. We have double-checked the manuscript to ensure the language reflects this. To align with the reviewer’s suggestion, we specifically revised the Introduction (line 100) to describe our work as providing "key system contributions" rather than "innovations." We believe this accurately frames the work as a comprehensive tooling platform.

Reviewer #2

We appreciate Reviewer #2's positive assessment and their recognition of EdgeVolution's contribution to closing the gap between AI breakthroughs and real-world implementation.

Comment 1: "One question then arrises, whether this approach can also further enhance the efficiency of hardware neural networks and neuromorphic systems applied in edge-computing and in-sensor computing devices, and how this can be envisioned in the future?"

Response: We thank the reviewer for this forward-looking question. We agree that the hardware-agnostic nature of EdgeVolution makes it a strong candidate for optimizing hardware neural networks and neuromorphic systems.

While our current work focuses on standard microcontrollers, the framework's modular design allows for the integration of customizable neuromorphic chips (such as Innatera's Pulsar) as execution targets. By adapting the fitness function to capture measuring specific neuromorphic metrics (e.g., spike rates or analog power consumption), EdgeVolution could automate the co-design of these next-generation architectures just as it currently does for standard CNNs. We have expanded the Future Work section (lines 468-473) to explicitly outline this promising research direction.

Reviewer #3

We are grateful to Reviewer #3 for their positive feedback and for acknowledging the utility of our multi-task demonstrations. We have addressed the specific questions below.

Comment 1: "Details about the neural network model being used for each task is missing."

Response: We appreciate the reviewer's query regarding the model details. As our framework is designed for Neural Architecture Search (NAS), the study does not rely on a single, fixed neural network architecture. Instead, the framework dynamically generates and evaluates thousands of unique models derived from a specific Search Space.

The exact specifications of these potential models are detailed in the manuscript:

1. Search Space Definition: The components used to construct the models (e.g., layers, kernel sizes, operations) are described in Section 4.5 (Methods) and fully listed in the Appendix (Search Space).
2. Optimized Architectures: The structural properties of the resulting optimized models (e.g., depth, layer ordering) are analyzed in the Results section (specifically Figures 2d, 3d-e, and 4d-e).
3. Baseline Model: For comparison, we utilize EfficientNetB0 as the fixed baseline architecture.

Comments 2, 3 and 7: "It would be useful to add details about the evolutionary optimization in EdgeVolution and impacts of mutation and cross-over fractions in your evaluation."; "How does your evolutionary optimization method compare with other EAs such as LEAP and MENNDL?" and "Why does the population size decrease over generations, as shown in the supplementary document appendix E?"

Response: We thank the reviewer for these interconnected questions regarding the design and dynamics of our evolutionary algorithm. The mechanics of our optimization are detailed in

Section 4.1 and Section 4.5. We employ a dynamic parameter decay strategy, where both the population size and mutation rate decrease over time (visualized in Appendix G, Figure G1).

- Rationale: This design mimics an annealing process. We begin with a large population and a high mutation rate to maximize exploration of the search space and maintain diversity. As the search progresses, we reduce these parameters to focus on exploitation, refining the best-performing candidates.
- Computational Efficiency: Reducing the population size in later generations is also a practical necessity. It allows us to allocate computational resources effectively, keeping the total search time feasible (approx. 4,600 evaluations) while ensuring the algorithm converges on an optimal solution.

Our primary contribution is the system-level integration of hardware-in-the-loop (HITL) for microcontrollers, rather than the proposal of a novel evolutionary algorithm. Consequently, we utilized a lightweight, custom "bare-metal" genetic algorithm to validate the pipeline. While established libraries like LEAP or MENNDL offer sophisticated features, our current implementation is sufficient to demonstrate the efficacy of the HITL approach. Importantly, as noted in Section 2.1, the framework's modularity allows for the seamless integration of other NAS algorithms (e.g., NSGA-II or LEAP), which is a planned feature for future releases. Nevertheless, we changed the manuscript to acknowledge different evolutionary algorithms and cite these, i.e. LEAP and MENNDL, accordingly (line 131).

Comment 4: "Why does Mel scaling not perform as well or better than FFT based preprocessing in the audio application?"

Response: We thank the reviewer for highlighting this interesting finding. As noted in the manuscript (Section 2.3), this result indeed contradicts standard conventions in speech processing. Our analysis indicates that for the extremely small, resource-constrained models required on microcontrollers, the dimensionality reduction and information loss inherent to Mel-scaling are disadvantageous. While Mel-scaling aligns features with human perception, it compresses frequency bins. The evolutionary search discovered that for these specific tiny architectures, providing the raw, higher-resolution STFT features yielded better discriminative power than the compressed Mel features. This is supported by our data in Figure 3f (which shows the algorithm actively discarding Mel-scaling over generations) and Figure 3g (which shows a statistically significant performance penalty for models using it). This underscores the value of NAS in uncovering hardware-specific design choices that differ from established "best" practices.

Comment 5: "For CIFAR-10, you mention using 90% of the dataset for training and 10% for validation. Does the EA optimize for validation set? How is the test set defined?"

Response: We thank the reviewer for asking for this clarification regarding the data splitting strategy.

- Data Splitting: We utilized the standard CIFAR-10 configuration provided by TensorFlow Datasets (TFDS), which consists of 50,000 training images and 10,000 test images. For the search process, we split the 50,000 training images into a 90% partition for network training and a 10% partition for validation. The official 10,000-sample test set was kept strictly separate.
- Optimization Target: Yes, the Evolutionary Algorithm (EA) optimizes for the validation accuracy (calculated on the 10% split from the training data). This ensures that the fitness function rewards generalization capabilities without overfitting to the final test set.
- Test Set: When the "test set" is mentioned, it refers exclusively to the official, held-out 10,000 images, which are used only for the final benchmarking of the best-found architectures.

We have updated Section 4.4 (Cifar-10) to explicitly describe this 90/10 split of the training set and the reservation of the official test set (lines 650-655).

Comment 6: "In your fitness function in equation (1), what are the values for wM and wE?"

Response: We thank the reviewer for this question. As detailed in the Discussion section and the configuration listing in Appendix D, the specific weights used for all experiments in this study were:

- w_A = 0.7 (Accuracy)
- w_M = 0.1 (ROM usage)
- w_E = 0.2 (Energy consumption)

These values were selected empirically to prioritize classification accuracy while maintaining sufficient hardware impact to optimize for resource efficiency.

We believe that the revisions have substantially strengthened the manuscript and have addressed all the concerns raised by the reviewers. We look forward to your evaluation of the revised paper and hope that it is now suitable for publication in *Communications Engineering*.

Sincerely,
René Groh

René Groh
Department Artificial Intelligence in Biomedical Engineering
rene.groh@fau.de

28.03.2026

The Editorial Office
Communications Engineering

Subject: Rebuttal Letter for Manuscript ID: COMMSENG-25-0589-T - "EdgeVolution: Democratizing Multi-Objective Neural Architecture Search and End-to-End Deployment on Microcontrollers"

Dear Editors,

Thank you for managing the review process for our manuscript and for providing us with the reviewers' insightful feedback. We also would like to thank the reviewers for their time and constructive comments, which have helped us to further improve the quality of our paper and expanded the scope and usability to the community.

We have carefully considered all the points raised by the reviewers and have revised the manuscript accordingly. Below, we provide a point-by-point response to each of the reviewers' remaining comments. All changes in the revised manuscript have been highlighted for your convenience.

Reviewer #1

We thank Reviewer #1 for their thorough re-evaluation. We fully agree with their final suggestions and have therefore integrated all proposed features (in total four) into the EdgeVolution framework, alongside comprehensive code documentation. All of the following results were obtained using the Speech Commands dataset:

Comment 1: "Possibility of training surrogate models for accuracy"

Response: We added the option to train a surrogate model (Random Forest or Gaussian Process) to predict the validation accuracy of a given neural network architecture.

To evaluate this capability, we trained a Random Forest (100 estimators) on architecture encodings from generation 1 (N=100) and ran the search in evaluation mode, where the surrogate predicts accuracy for every individual, but no training is skipped, preserving ground-truth labels for all 1,000 architectures across 30 generations. Supplementary Fig. D(a) shows predicted vs. actual accuracy across all generations (Pearson r = 0.65, MAE = 0.14), demonstrating that the surrogate captures the overall accuracy landscape. The surrogate's predictive performance improves as more training data accumulates (Pearson r increases from

0.50 in generations 2-15 to 0.62 in generations 16-30). In production mode, the surrogate skips training for individuals whose predicted accuracy falls below a configurable confidence threshold, while an exploration ratio (default 20%) ensures a fraction of low-predicted individuals are still trained to prevent self-reinforcing bias. Based on the evaluation-mode data, applying the default threshold (0.50) would have allowed skipping approximately 25% of training runs without affecting the final best architecture found.

Comment 2: "Possibility of building a lookup table for a given hardware target"

Response: We implemented a hardware lookup table (LUT) system that allows users to profile a target microcontroller once and then use the collected data to predict hardware metrics (energy consumption, inference time) for new architectures without requiring on-device measurement during the search.

The LUT is built by training Random Forest models (100 estimators) on architecture encodings paired with measured hardware metrics from an initial profiling run (N=1,000 architectures on the nRF52840). To validate the approach, we ran a full 30-generation search using the LUT for hardware prediction while still performing actual on-device measurements for comparison. Supplementary Fig. E shows the LUT predictions vs. actual measurements for energy consumption (Pearson r = 0.59, N=943) and inference time (Pearson r = 0.60, N=943). While the LUT predictions are less accurate than direct measurements, they still provide a useful approximation that eliminates the need for a physical hardware connection during the search, enabling faster iteration and remote execution after the initial LUT build.

Comment 3: "Possibility of training a surrogate model for a given hardware target"

Response: Analogous to the accuracy surrogate (Comment 1), we implemented a surrogate model for predicting hardware energy consumption.

A Random Forest is trained on architecture encodings paired with measured energy values and updated incrementally as new measurements become available. We ran a full 30-generation evaluation where the surrogate predicts energy for every individual while actual measurements are still performed for validation. Supplementary Fig. D(b) shows predicted vs. actual energy consumption (Pearson r = 0.72, MAE = 6.0 mJ, N=770). Notably, the surrogate's correlation improves substantially in later generations (reaching r > 0.95 in generations 20 and 30), demonstrating that the model becomes increasingly reliable as training data accumulates. This approach can replace or complement the hardware LUT (Comment 2) by learning directly from ongoing search data rather than requiring an upfront profiling run.

The additions resulting from Comment 1, Comment 2 and Comment 3 are mentioned in the main manuscript in Section 2.3 (lines 331-338) and Section 3 (lines 461-464).

Comment 4: "Exploring multiple search strategies."

Response: To demonstrate EdgeVolution's modularity, we implemented five search strategies as selectable parameters: our custom Genetic Algorithm, Regularized Evolution, NSGA-II, Bayesian Optimization, and Random Search.

All strategies share the same evaluation pipeline and search space, differing only in how candidate architectures are proposed. We ran each strategy for 30 generations. Supplementary Fig. B(a) shows the convergence of the best validation accuracy over generations. Our own genetic algorithm (GA) implementation achieves the highest final accuracy (0.909), followed by Regularized Evolution (0.850), NSGA-II (0.835), Bayesian Optimization (0.787), and Random Search (0.758). Fig. B(b) shows the explored architecture space (accuracy vs. TFLite model size), illustrating how each strategy samples different regions of the search space. Our own GA explores a wider range of model sizes and finds higher-accuracy architectures across the size spectrum. All strategies are available as configuration options and can be combined with the hardware-in-the-loop evaluation, surrogate models, or LUT described above. We mention the newly implemented search strategies in the main manuscript in Section 2.3 (lines 320-326) and Section 4.1 (lines 532-535).

Reviewer #3

We thank Reviewer #3 for the positive feedback. We are happy to answer here to the one remaining comment:

Comment 1: "It would be good to include the explanation for Mel scaling versus STFT in the paper as well in the Section 2.3 on Keyword spotting application."

Response: We thank the reviewer for this suggestion. We have added a brief explanation of the short-time Fourier transform (STFT) and Mel scaling to Section 2.3 (lines 275-283) to make the preprocessing pipeline more accessible to readers unfamiliar with audio signal processing. The added text clarifies that the STFT converts the raw audio waveform into a time-frequency representation by applying the Fourier transform over overlapping windows (controlled by the FFT window size and hop length), and that Mel scaling subsequently re-maps the linear frequency axis onto the perceptually motivated Mel scale, which compresses higher frequencies where human hearing is less sensitive. We also clarified (lines 283-291) why both are included as searchable parameters rather than fixed design choices: on microcontrollers, the resolution of these transforms directly affects RAM usage and tensor sizes, making the optimal configuration hardware-dependent.

We have comprehensively and successfully addressed every single concern raised by the reviewers. Given these extensive additions, the conclusive data provided, and resolving all outstanding queries, we believe the manuscript is now fully ready for publication in *Communications Engineering*.

Sincerely,
René Groh
